# The Phosphatomes of the Multicellular Myxobacteria *Myxococcus xanthus* and *Sorangium cellulosum* in Comparison with Other Prokaryotic Genomes

**DOI:** 10.1371/journal.pone.0011164

**Published:** 2010-06-17

**Authors:** Anke Treuner-Lange

**Affiliations:** Institut für Mikrobiologie und Molekularbiologie, Justus-Liebig-Universität, Giessen, Germany; University College Dublin, Ireland

## Abstract

**Background:**

Analysis of the complete genomes from the multicellular myxobacteria *Myxococcus xanthus* and *Sorangium cellulosum* identified the highest number of eukaryotic-like protein kinases (ELKs) compared to all other genomes analyzed. High numbers of protein phosphatases (PPs) could therefore be anticipated, as reversible protein phosphorylation is a major regulation mechanism of fundamental biological processes.

**Methodology:**

Here we report an intensive analysis of the phosphatomes of *M. xanthus* and *S. cellulosum* in which we constructed phylogenetic trees to position these sequences relative to PPs from other prokaryotic organisms.

**Principal Findings:**

Predominant observations were: (i) *M. xanthus* and *S. cellulosum* possess predominantly Ser/Thr PPs; (ii) *S. cellulosum* encodes the highest number of PP2c-type phosphatases so far reported for a prokaryotic organism; (iii) in contrast to *M. xanthus* only *S. cellulosum* encodes high numbers of SpoIIE-like PPs; (iv) there is a significant lack of synteny among *M. xanthus* and *S. cellulosum*, and (v) the degree of co-organization between kinase and phosphatase genes is extremely low in these myxobacterial genomes.

**Conclusions:**

We conclude that there has been a greater expansion of ELKs than PPs in multicellular myxobacteria.

## Introduction

All living cells must sense changes in their environment and respond appropriately by adjusting cellular processes. Reversible protein phosphorylation is a widespread and major mechanism for such cellular process regulation. The phosphorylation and dephosphorylation of proteins causes conformational changes that impinge upon the ability of the protein to interact with ligands. The chemistry underlying signal transduction processes either entails the formation of high-energy phosphoamino acids like phosphohistidine and phosphoaspartate or results in the formation of phosphoesters of serine, threonine and tyrosine residues. Formation of phosphoesters is catalyzed by protein kinases which act either on both serine and threonine, or on tyrosine, or on all three amino acids (dual-specificity kinases). The eukaryotic protein kinase (ePK) domain mediates the majority of signalling and coordination of complex events in eukaryotes. The identification of ePK-like protein kinases (ELKs) in genomes of bacteria and *Achaea* showing in the area of the catalytic sites high levels of primary structural homology, led to the suggestion that the corresponding ancestral genes arose at the level of prokaryotes and Archaea [1], [2], [3].

The Myxobacteria are a remarkable group of δ-Proteobacteria with a complex multicellular developmental program (for review [4]). Genome analyses revealed that ELKs are highly represented in the myxobacteria [5]. *M. xanthus*, in which the first prokaryotic ELK was identified, contains a total of 99 ELKs [5], [6]. The genome of the myxobacterium *Sorangium cellulosum* So ce56 encodes 317 ELKs [5], [7], by far the largest number of ELKs genes detected in a prokaryotic genome.

In *M. xanthus*, the best-studied myxobacterium in regard to multicellular development and fruiting body formation, at least one third of the ELKs are necessary for appropriate development (for review [8]).

The phosphorylation level of a given proteome reflects not only the activities of protein kinases, but also of opposing protein phosphatases (PPs), which cleave the monophosphate esters from phosphorylated serine, threonine and/or tyrosine residues. In contrast to the ELKs, the PPs in eukaryotes and bacteria show a much more diverse relationship [9], [10], [11], [12]. The known bacterial PPs belong to the following three major families:

- The PPM-family of metal-dependent serine/threonine PPs,
- the PPP-family of serine/threonine PPs,
- the PTP-family of tyrosine-specific PPs including dual-specificity PTPs, low molecular weight protein tyrosine phosphatases (low MW PTPs), and PTPZ-like protein tyrosine phosphatases [1], [13], [14].

To date, the highest number of bacterial PPs within a genome was reported for *Streptomyces coelicolor* A3(2) and *S. avermitilis*. The number of 55 PPs even exceeds the number of ELKs (37 resp. 33) found in these genomes [15].

As mentioned, *M. xanthus* and *S. cellulosum* harbors 3-10 times more ELKs. Therefore the question arose how many PPs are encoded in the genomes of *M. xanthus* and *S. cellulosum* and what their role is.

Pph1 was the first PPM PP identified from *M. xanthus*. The phenotype of a *M. xanthus* DZ2 strain bearing an insertion in the *pph1* gene indicated the protein is involved in regulating vegetative growth and the development of multicellular fruiting bodies [16]. Measurements of protein phosphatase activity in protein extracts from wildtype and from the DZ2*pph1* (DZ4314) mutant indicated the existence of additional PPM PPs in the organism [16]. Deletion of the *pph2* gene, which encodes a PPP PP from *M. xanthus*, indicated that the protein is important for fruiting body formation and sporulation [17]. The genome of *M. xanthus* was reported to encode 34 PPs (5 PPMs, 4 PTPs, 25 PPPs) [8]. However, some proteins of that proposed PPP group have been characterized as phosphodiesterases [18], [19] and additional signature domains of bacterial PPP PPs have been defined [10] suggesting that the phosphatome of *M. xanthus* is significantly smaller. With the complete genome of *S. cellulosum* the phosphatomes of these two species can be compared and the question of whether a certain type of PP is overrepresented in those myxobacterial genomes encoding high numbers of ELKs, can be answered.

Both organisms belong to the order *Myxococcales* but to two different suborders, which members are different in regard to 16S rRNA, physiology and fruiting body formation [4], [20]. An intensive bioinformatic analysis of putative PPs in the genome of *M. xanthus* DK1622 and *S. cellulosum* So ce56 was carried out. The phosphatomes of these two multicellular myxobacteria were then compared to 66 genomes of the COG database (http://www.ncbi.nlm.nih.gov/COG/) as well as to phosphatomes of non-multicellular myxobacteria, such as *Anaeromyxobacter dehalogenans* 2CP-C, and two non-myxobacterial organisms such as *Streptomyces coelicolor* A3(2) and *S. avermitilis*, which also undergo morphological differentiation processes [21].

Protein kinase to protein phosphatase ratios of 1-3/1 were observed in eukaryotic, archaeal as well as prokaryotic organisms [3], [8], [22], [23]. If such a ratio applies also to *S. cellulosum*, it could possess approximately 100–170 PPs.

## Results

Our genome comparison set of 72 genomes includes 64 genomes (3 Eukarya, 13 Archaea, 48 Bacteria) of the 66 genome COG database and in addition six myxobacterial and two actinobacterial genomes (Table S1). In the phylogenetic trees, sequences from up to 55 species were used (only one species per genus, Table 1).

**Table 1:** List of organisms and phyla used in this study.

SK	phylum	code	*organism*
B	*Actinobacteria*	Cgl	*Corynebacterium glutamicum*
B	*Actinobacteria*	Mtu	*Mycobacterium tuberculosis H37Rv*
B	*Actinobacteria*	Sco	*Streptomyces coelicolor A3(2)*
B	*Aquificae*	Aae	*Aquifex aeolicus*
B	*Chlamydiae*	Ctr	*Chlamydia trachomatis*
B	*Cyanobacteria*	Nos	*Nostoc sp. PCC 7120*
B	*Cyanobacteria*	Syn	*Synechocystis*
B	*Deinococcus-Thermus*	Dra	*Deinococcus radiodurans*
B	*Firmicutes*	Bsu	*Bacillus subtilis*
B	*Firmicutes*	Cac	*Clostridium acetobutylicum*
B	*Firmicutes*	Lin	*Listeria innocua*
B	*Firmicutes*	Lla	*Lactococcus lactis*
B	*Firmicutes*	Mpn	*Mycoplasma pneumoniae*
B	*Firmicutes*	Sau	*Staphylococcus aureus N315*
B	*Firmicutes*	Spn	*Streptococcus pneumoniae TIGR4*
B	*Firmicutes*	Uur	*Ureaplasma urealyticum*
B	*Fusobacteria*	Fnu	*Fusobacterium nucleatum*
B	*Proteobacteria*	Adeh	*Anaeromyxobacter dehalogenans 2CP-C*
B	*Proteobacteria*	Atu	*Agrobacterium tumefaciens str. C58*
B	*Proteobacteria*	Bme	*Brucella melitensis*
B	*Proteobacteria*	Buc	*Buchnera sp. APS*
B	*Proteobacteria*	Ccr	*Caulobacter vibrioides*
B	*Proteobacteria*	Cje	*Campylobacter jejuni*
B	*Proteobacteria*	EcZ	*Escherichia coli O157:H7 EDL933*
B	*Proteobacteria*	Hin	*Haemophilus influenzae*
B	*Proteobacteria*	Hpy	*Helicobacter pylori 26695*
B	*Proteobacteria*	Mlo	*Mesorhizobium loti*
B	*Proteobacteria*	Mxan	*Myxococcus xanthus DK1622*
B	*Proteobacteria*	Nme	*Neisseria meningitidis MC58*
B	*Proteobacteria*	Pae	*Pseudomonas aeruginosa*
B	*Proteobacteria*	Pmu	*Pasteurella multocida*
B	*Proteobacteria*	Rco	*Rickettsia conorii*
B	*Proteobacteria*	Rso	*Ralstonia solanacearum*
B	*Proteobacteria*	Sce	*Sorangium cellulosum So ce56*
B	*Proteobacteria*	Sme	*Sinorhizobium meliloti*
B	*Proteobacteria*	Sty	*Salmonella typhimurium LT2*
B	*Proteobacteria*	Vch	*Vibrio cholerae*
B	*Proteobacteria*	Xfa	*Xylella fastidiosa 9a5c*
B	*Proteobacteria*	Ype	*Yersinia pestis*
B	*Spirochaetes*	Tpa	*Treponema pallidum*
B	*Thermotogae*	Tma	*Thermotoga maritima*
A	*Crenarchaeota*	Ape	*Aeropyrum pernix*
A	*Crenarchaeota*	Pya	*Pyrobaculum aerophilum*
A	*Crenarchaeota*	Sso	*Sulfolobus solfataricus*
A	*Euryarchaeota*	Afu	*Archaeoglobus fulgidus*
A	*Euryarchaeota*	Hbs	*Halobacterium sp. NRC-1*
A	*Euryarchaeota*	Mac	*Methanosarcina acetivorans str.C2A*
A	*Euryarchaeota*	Mja	*Methanococcus jannaschii*
A	*Euryarchaeota*	Mka	*Methanopyrus kandleri AV19*
A	*Euryarchaeota*	Mth	*Methanothermobacter thermautotrophicus*
A	*Euryarchaeota*	Pho	*Pyrococcus horikoshii*
A	*Euryarchaeota*	Tvo	*Thermoplasma volcanium*
E	*Ascomycota*	Scer	*Saccharomyces cerevisiae*
E	*Ascomycota*	Spo	*Schizosaccharomyces pombe*
E	*Microsporidia*	Ecu	*Encephalitozoon cuniculi*

Abb = abbreviation; SK = superkingdom; B = Bacteria; E = Eukaryota; A = Archaea. The 66 genome COG database comprises a few similar species from on genus. In this study we restricted the selection to one species per genus. The complete list of organisms can be seen in Table S1.

### Predominantly serine/threonine specific bacterial PPs

This group of PPs splits into PPM-family and PPP-family members (Table 2). The PPM PPs belong to the PP2c superfamily. Two different pfam profiles (PF00481 and PF07228) can be found in this family, indicating a rather divergent family. However, although it was obviously not possible to create a single HMM that detects all examples of this family the members are assumed to have arisen from a single evolutionary origin [24]. The PPM family proteins are assigned to two different clusters of orthologous groups (COG0631 and COG2208). A COG group consists of individual orthologous proteins which typically have the same function, allowing transfer of functional information from one member to an entire COG [25]. Whereas, the pfam profiles define only certain domains of a given protein a COG assignment of a protein gives some additional information about the degree of sequence conservation of the full-length protein, therefore we provide in this study COG as well as pfam data.

**Table 2:** Groups of predominantly serine/threonine specific bacterial PPs in regard to specific pfam domains and COG assignments.

metal-dependent PPs (PPMs)		phosphoprotein phosphatases (PPPs)		
cl00120 PP2Cc superfamily		part of the calcineurin-like superfamily		
pfam00481	pfam07228	pfam00149		
COG0631	COG2208	COG0639		other COGs
PP2c-type PPs	SpoIIE-like PPs	bacterial PPPs	Diadenosinetetraphosphatases	phosphodiesterases, nucleotidases, exonucleases

According to [10], [12], [15] and the COG and pfam database.

In the following the terms PP2c-type PPs (PF00481, COG0631) and SpoIIE-like PPs (PF07228; COG2208) are used to address issues concerning these two subgroups of the PPM family (Table 2).

### a) The PP2c-type PPs

**Figure 1:**
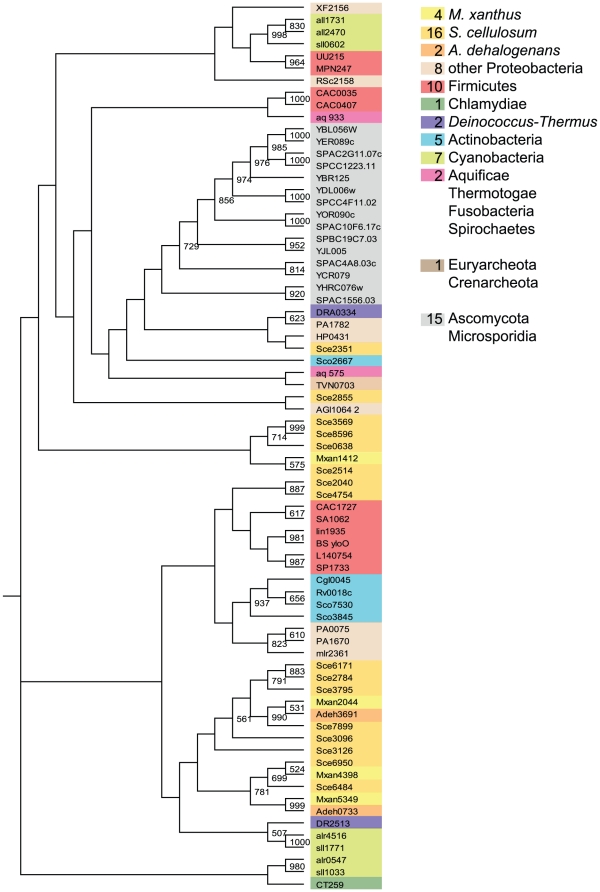
The PP2c-type PPs derived bootstrapped tree (1000 replicates). Numbers of proteins in the three myxobacterial species as well as in other phyla are given in the colored boxes. The same color code was used to label the sequences within the tree. Only bootstrap values of 50% and above are shown.

Our analysis revealed that the genome of *S. cellulosum* encodes 16 PP2c-type PPs. This is the highest number so far reported for a bacterium, and exceeds even the number of PP2c-type PPs found in the two Ascomycota of our genome set (Fig. 1, Table S1). *M. xanthus* only harbors 4 PP2c-type PPs (Fig. 1, Table S1). Considering that *S. cellulosum* harbors 3.2 times more kinases than *M. xanthus* this high number supports the hypothesis that these proteins are counteracting the activity of ELKs in myxobacteria. Also, there is a significant correlation of PP2c-type PP and ELK harboring organism (Table S1). This hypothesis is partially supported for *S. cellulosum* as six co-organizations of ELK and PP2c-type PP encoding genes were found. Similar co-organizations were not found in *M. xanthus* and *A. dehalogenans*.

However, the fact that *P. aeruginosa* and *Synechocystis* sp. each contain three PP2c-type PPs while harboring only 7 and 9 ELKs, respectively (Table S1), also suggests that the myxobacterial PP2c-type PPs did not expand in number to the same degree as the myxobacterial ELKs.

The bootstrap tree shows that the PP2c-type PPs cluster mainly according to the species of origin (Fig. 1), as clades with rather eukaryotic, cyanobacterial, actinobacterial and myxobacterial sequences did form. To identify gene products which originated from a common ancestor we looked for myxobacterial orthologs (BLASTP E-value ≤10^−10^) using reciprocal Blastp analyses. The three suggested groups of orthologs cluster tightly together (mxan4398∶sce6484, mxan2044∶sce7899∶adeh3691, and mxan5349∶adeh0733). The tree indicates that the elevated number of PP2c-type PPs in *S. cellulosum* is mostly the result of gene duplication after speciation. However, the existence of genes like sce2351 and sce2855 might be the result of lateral gene transfer.

A majority of the PP2c-type PPs in this study contain only the catalytic PP2c-type domain, but some have additional N- or C-terminal extensions, transmembrane domains and/or other catalytic domains. Here we only would like to mention that three myxobacterial PP2c-type PPs (Mxan4398, sce2855, sce6950) have an additional C-terminal cyclic nucleotide-monophosphate binding domain (cNMP), which represents, according to the cdart database [26], a domain combination not found outside the *Myxococcales*. This cNMP-domain suggests that the activity of these PPs is regulated by cyclic nucleotides such as cAMP or cGMP (SMART accession SM00100).

### b) SpoIIE-like PPs

**Figure 2:**
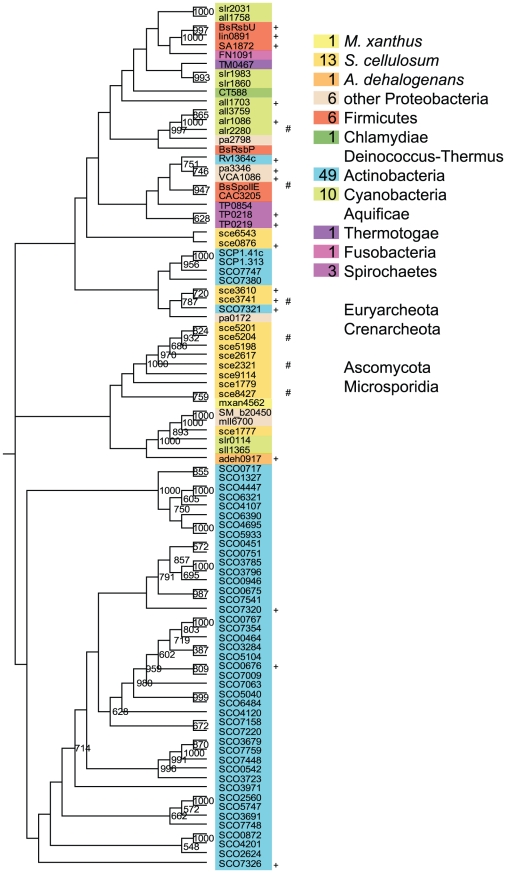
The SpoIIE-like PPs derived bootstrapped tree (1000 replicates). Numbers of proteins in the three myxobacterial species as well as in other phyla are given in the colored boxes. The same color code was used to label the sequences within the tree. Only bootstrap values of 50% and above are shown. The + indicates the proteins to be part of a putative RsbRST cluster, the # indicates and ELK-encoding genes in the genetic neighborhood (max. 5 gene distance).

Studying the group of SpoIIE-like PPs from myxobacteria reveals a significant difference between *M. xanthus* and *A. dehalogenans* on one side, and *S. cellulosum* on the other. While *M. xanthus* and *A. dehalogenans* possess each only one SpoIIE-like PP, *S. cellulosum* harbours thirteen such phosphatases (Figure 2, Table S1). The majority of these myxobacterial SpoIIE-like PPs cluster in a central group, together with two out 10 cyanobacterial and two out of six proteobacterial proteins. This central cluster splits into two clades. The top clade includes 8 proteins only from *S. cellulosum*, most of which contain a periplasmic binding domain (PBD, pfam00532). This domain organization is unique for *S. cellulosum* in the used genome-set and also the cdart tool finds only two similar proteins one of a cyanobacterial and one delta-proteobacterial organism. The clade expansion seems to be the result of gene duplication after speciation. However, four additional sce proteins (sce6543, sce0876, sce3610, sce3741) which cluster in the top group and show higher similarity to some actinobacterial proteins could be inherited by *S. cellulosum* by lateral gene transfer.

Only one group of myxobacterial orthologous proteins could be identified (sce8427∶mxan4562∶adeh0917). The latter proteins both have an additional HAMP domain (pfam00672) and represent the only type of SpoII-like PP in *M. xanthus* and *A. dehalogenans*.

In total *S. cellulosum* has five different types of these SpoII-likePPs: in addition to the PBD-domain fusion, HAMP (pfam00672; sce1777), FHA (pfam00498; sce0876) and GAF (pfam01590; sce6543) domains are fused to these proteins. The fusion of the SpoIIE-like PP domain with additional domains is not unusual and also known from other bacteria [15], [27].

**Figure 3:**
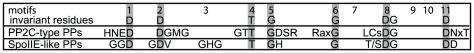
Comparison of the signature sequence of PPMs [**15**], with those found in the myxobacterial PP2C-type and SpoIIE-like PPs. The single-letter amino acid code is used.

Proteins like SpoIIE, RsbV, RsbS and RsbU are involved in regulating sigma-factors in so called partner-switching modules [28], [29]. In general, these protein act on anti-anti-sigma factors (COG1366) and modulate the anti-sigma factors (COG2172), which represent non-ELK-like protein kinases [28]. Because these proteins are often encoded by conserved gene clusters, the name RsbRST module was defined [28]. Therefore, although a significant expansion of SpoIIE-like PPs occurred in *S. cellulosum* it is not clear whether these proteins are involved in opposing ELKs. Especially, because in parallel the number of non-ELK-like protein kinases (COG2172) and anti anti-sigma factors (COG1366) also increased in *S. cellulosum*. In total we found 71 sce proteins assigned to COG 1366. This represents the highest number of these proteins in a single prokaryotic genome to the best of our knowledge. Interestingly, in *S. cellulosum* these proteins can be found fused to ELKs suggesting an ELK-linked function of this domain in this organism. In order to substantiate this assumption, we analysed if in the neighbourhood of the SpoIIE-like PP encoding genes we could find ELK- (COG0515) or non ELK-(COG2172) encoding genes. Only a few SpoIIE-type PP encoding genes are indeed genetically linked to kinase encoding genes (ELK = # and/or non-ELK = + in Fig. 3).

SpoIIE-like PPs are abundant in *Sorangium* but not more abundant than in *Streptomyces* genomes (Fig. 3). It was hypothesized that in *Streptomyces* these phosphatases are relatively recent additions from a eukaryotic source [15], [30]. However, a phylogenomic study of SpoII-like PPs suggests that the regulation by partner switching mechanisms was invented rather early in evolution [27].

Fig. 3 indicates that the sco as well as the sce cluster originated similarly but evolved independently.

The PP2c-type and SpoIIE-like PPs belong to the PP2c superfamily, indicated by the fact that they both share a catalytic domain consisting of eleven sequence motifs just like eukaryotic PP2Cs [1], [30], [31]. In multiple sequence alignments several conserved residues in the myxobacterial PPMs can be found (Figure S1 and S2). Although the members of these subfamilies share conserved amino acids they differ particularly when comparing neighboring residues (Fig. 3).

### PPP-phosphatases

To distinguish between genuine PPP-phosphatases and related non-phosphatase enyzmes we looked for protein with the pfam profile PF00149, but especially looked for the COG groups the proteins were assigned to. This is important because PPP-PPs belong to the calcineurin-like superfamily which consists of enzymes with diverse functions [32]. Members of this superfamily hydrolyze a wide variety of protein and nucleotide substrates, including PPs, nucleotidases, sphingomyelin phosphodiesterases, and 2′,3′-cAMP phosphodiesterases, as well as nucleases such as bacterial SbcD [32], [33](Table 2).

Therefore not every protein with the PF00149 profile is necessarily a protein phosphatase. Because a COG assignment in not only based on the catalytic domain but also on the protein regions outside the catalytic domains, the 96 myxobacterial proteins with the PF00149 profile, belong to 12 different COG categories and nine have not been assigned a COG. Of these twelve COG groups only the COG0639 group comprises proteins with experimentally proven protein phosphatase (PPP-like activity) and/or diadenosine tetraphosphatase activity (ApaH-like activity) [17], [34], [35], [36], [37]. An extensive phylogenetic analysis on bacterial, archaeal and eukaryotic PPPs was performed before completion of the *M. xanthus* genome, and identified one myxobacterial PPP (mxan5467), as a *Shewanella*-like phosphatase (Shelph) [10]. Therefore we concentrate in this paper on the myxobacterial proteins. PPP-PPs were so far known for the three conserved motifs (GDXHG/GDXXDRG/GNHE) [15]. A previously unrecognized (I/L/V)D(S/T)G motif has later been found in all bacterial and also” bacterial-like” eukaryotic PPPs [10], which could be used to distinguish PPP-like proteins from ApaH-like proteins.

PPP-PPs are abundant in myxobacteria, with no significant differences in regard to total numbers within the three species (Table S1). With seven such proteins *S. cellulosum* harbors slightly more than *M. xanthus* and *A. dehalogenans* each harbouring five.

**Figure 4:**
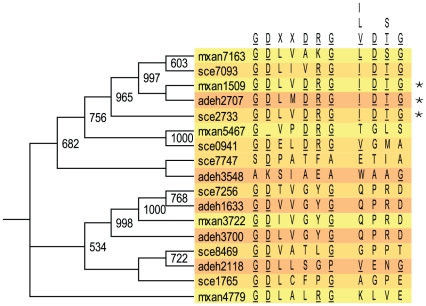
The myxobacterial PPP derived bootstrapped tree (1000 replicates). Only bootstrap values of 50% and above are shown. Next to the tree an alignment is shown in the areas of the GDXXDRG motif [15] and the newly indentified (I/L/V)D(S/T)G motif [10]. The star (*) labels those sequences which match 100% with the consensus.

In Fig. 4 a bootstrap tree of these seventeen myxobacterial proteins is combined with the alignments of the GDXXDRG and the (I/L/V)D(S/T)G motifs. Only three proteins have all the important residues conserved (*, Fig. 4). However, we are not convinced that proteins with altered residues within those motifs are not catalytically active, as the known Pph2 PP from *M. xanthus* (mxan4779) has been shown to dephosphorylate phosphopeptides [17].

Interestingly, although this group of PPs is supposed to act as Ser/Thr PPs, some of these proteins can remove phosphate from phosphotyrosine [17], [35], [36], [37]. Therefore, the myxobacterial COG0639 proteins might represent not only serine/threonine PPs but also tyrosine phosphatases and, further, diadenosine tetraphosphatase activity can not be ruled out. The number of PPPs per myxobacterial genome is not unusually high. Other bacteria such as *Deinococcus radiodurans* comprises even more PPPs, and there is no correlation between abundance of ELK-encoding genes and PPP-encoding genes in the used genome-set (Table S1).

### Phosphotyrosine-PPs

The group of predominantly tyrosine-specific PPs includes PTPs, dual-specificity (DSP) PTPs, low molecular weight protein tyrosine phosphatases (low MW PTPs), and PTPZ-like protein tyrosine phosphatases [1], [13], [14]. Bacterial phosphotyrosine-PPs are due to their primary sequences assigned to six COG groups (Table 3). In the myxobacterial genomes proteins assigned to four of these COG groups could be identified (Table 3). As already observed for the PPPs, the number of predominantly tyrosine-specific PPs is not unusually high in comparison to the genomes of the COG database (Table 3, Table S1).

**Table 3:** Abundance of myxobacterial PTPs in contrast to the 66 genome COG database.

COG group	description	abbreviation	COG database	Mxan	Sce	Adeh
COG2453	Predicted protein-tyrosine phosphatase	PTP	27/17/6	3	3	1
COG0394	Protein-tyrosine-phosphatase	low MW PTPs	70/38/5	1	1	2
COG2365	Protein tyrosine/serine phosphatase	DSP PTPs	19/12/5		1	
COG4551	Predicted protein tyrosine phosphatase	PTP	4/4/1			
COG5350	Predicted protein tyrosine phosphatase	PTP	6/5/2			
COG4464	Capsular polysaccharide biosynthesis protein	PTPZ	7/5/2	1	1	

The three numbers in the COG database row represent the following: total number of proteins in the 66 genome COG database/total number of genomes in which those proteins were found/highest number of proteins per single bacterial genome.

**Figure 5:**
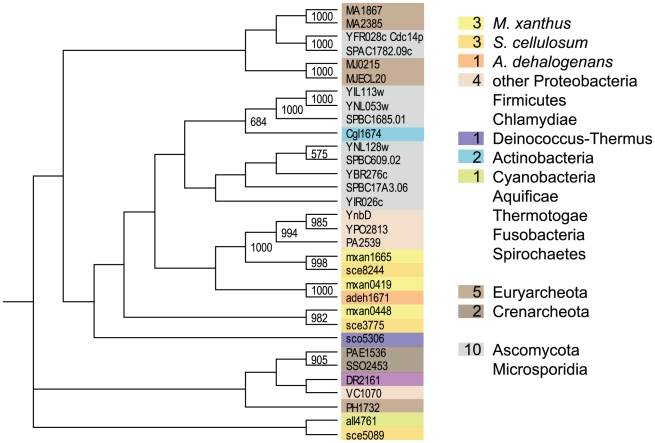
The PTP (only COG2453) derived bootstrapped tree (1000 replicates). Numbers of proteins in the three myxobacterial species as well as in other phyla are given in the colored boxes. The same color code was used to label the sequences within the tree. Only bootstrap values of 50% and above are shown.

The majority of the so far known COG2453 proteins are archaebacterial or eukaryotic (Fig. 5, Table S1). The well known Cdc14p protein from yeast, which is described as a dual specific PTP involved in cell cycle progression [38], belongs to that COG group. *M. xanthus* and *S. cellulosum* each contain three proteins of that kind, which indicates in contrast to *A. dehalogenans* a small expansion of these proteins in these two genomes. The boostrap tree indicates that these proteins group mostly according to the species of origin. The two ortholog groups mxan0419∶sce3775∶adeh1671 and mxan1665∶sce8244 cluster tightly together and are most similar to proteins from other proteobacteria. Only one protein from *S. cellulosum*, sce5089, clusters apart from the other proteins together with a cyanobacterial protein (Fig. 5). Unfortunately there is no experimental evidence found for any bacterial member of that group.

Several COG0394 proteins are encoded by genes, which are annotated as *arsC* genes. A significant structural similarity between arsenate reductase and bovine low molecular weight protein tyrosine phosphatase as well as a protein exhibiting both activities was reported earlier [1], [39], [40]. Therefore, without experimental evidence it is hard to speculate if the three orthologous myxobacterial proteins (mxan0575∶sce5614∶adeh1022) are indeed low MW PTPs.

*S. cellulosum* is unique within the myxobacteria in harboring one COG2365 protein. These proteins are more abundant in the Ascomycota, but can also be found in other actinobacteria and proteobacteria (Table S1).

We also looked for rhodanese domains (COG0607, PF0581) which are ubiquitous structural modules related to the catalytic subunit of Cdc25 phosphatase enzymes [41]. In eukaryotes Cdc25 phosphatases are suspected to be key players in cancer [42]. The myxobacterial genomes encode rhodanese proteins which are assigned to COG0607. However, none of them comprise the active-site motif CE[F/Y]SXXR that characterizes Cdc25 phosphatases [41].

A new group of PtpZ-like tyrosine phosphatases was recently defined [14]. So far, these proteins were named due to their regulatory function in exopolysaccharide production, and were therefore not easily recognized as phosphatases (Table 3) [14]. This group of proteins are well known from Gram-positive bacteria and belong to the polymerase and histidinol phosphatase family (PHP) [14]. It is interesting to find such proteins in *M. xanthus* (mxan0575) and *S. cellulosum* (sce5955), however neither are they suppose to counteract the activity of ELKs nor expanded in the genomes and therefore not further discussed in this paper.

### Synteny

**Figure 6:**
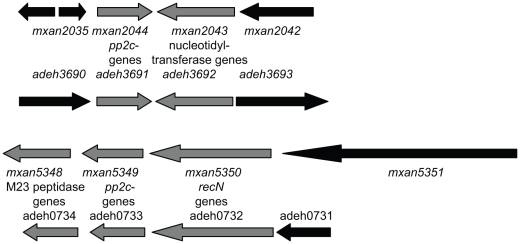
The only examples of local synteny within the PP-encoding genes are among PP2c-type encoding genes from *M. xanthus* and *A. dehalogenans*.

Poor global and local synteny was previously reported for the myxobacterial kinomes [5]. That is also true for the myxobacterial phosphatomes, for which only two examples of local synteny between *M. xanthus* and *A. dehalogenans* (mxan2044/adeh3691 and mxan5349/adeh0733) could be identified (Fig. 6). In both cases the corresponding genes encode for a PP2c-type PP.

### Ratios

The numbers of putative PPs revealed by this study and the corresponding ELKs/PP ratios are summarized in Table 4. The numbers clearly indicate that the number of PPs in the genomes of the multicellular myxobacteria did not increase in parallel to the expansion of the ELKs. The ELK/PP ratio is highest for *S. cellulosum* and *M. xanthus*. The expansion of ELK genes in these two myxobacterial genomes did not cause a significant expansion of PP genes The highest number of bacterial PPs is encoded by the genome of *S. coelicolor*, which is due to the already described expansion of SpoIIE-like PPs. While in *S. coelicolor* the number of PPs even exceeds the numbers of ELKs, the opposite trend is seen in *M. xanthus* and *S. cellulosum*.

**Table 4:** Numbers of ELKs and PPs and kinase/phosphatase ratios in selected genomes.

	ELKs	PP2c-type	SpoIIE-like	PPPs	PTPs	all PPs	ratio ELK/PP
*S. cellulosum*	315	16	13	7	5	41	7.7/1
*S. cerivisieae*	115	8	-	13	15	36	3.2/1
*M. xanthus*	96	4	1	5	4	14	6.9/1
*Nostoc sp. 7120*	50	4	5	5	5	19	2.6/1
*S. coelicolor*	35	3	46	2	6	57	0.6/1
*A. dehalogenans*	19	2	1	5	3	11	1.7/1
*Synechocystis*	9	3	3	1	1	8	1.1/1
*P. aeruginosa*	7	3	3	2	5	13	0.5/1
*B. subtilis*	5	1	3	1	3	8	0.6/1

Corresponding COG groups : ELKs (COG0515), PP2c-type (COG0631), SpoIIE-like (COG2208), PPPs (COG0639), PTPs (COG2453, 0394, 2365); see also Table S1.

## Discussion

*M. xanthus* and *S. cellulosum* are both multicellular myxobacteria, but differ significantly in regard to physiology, fruiting body formation and secondary metabolism [4], [7], [20], [43]. Phylogenetic analyses indicated two deep division lines within the Myxococcales, separating the suborders Cystobacterineae, Sorangineae and Nannocystineae [20], [44]. Exploring the genomes of the cellulolytic *S. cellulosum* (Sorangineae) and the bacteriolytic *M. xanthus* (Cystobacterineae) a high level of divergence was discovered [7]. The phosphatomes of these two organisms also differ in several regards and not one example of local synteny between these two organisms could be identified for any putative PP gene.

The ELKs, which are overrepresented within these two myxobacterial species, all belong to COG0515 and have been described in much detail [5], [8]. Here, we would like to comment on the anticipated specificity of these kinases, which in the past have been assumed due to their primary sequence and selected biochemical analyses to specifically phosphorylate serine and threonine residues [for review 8]. The catalytic domain of ePKs consists of 11 subdomains, comprising several conserved residues [45], [46]. Especially residues in the catalytic loop region (VIb) of ePKs determine the specificity of these kinases. The consensus D-L-K-P-E-N in this region is an indicator of serine/threonine specificity, whereas the protein-tyrosine kinase consensus is either D-L-R-A-A-N or D-L-A-A-R-N [45]. The myxobacterial ELKs also comprise these eleven subdomains, and are, based on some biochemical analyses and the fact that the majority comprises a lysine in the catalytic loop, believed to represent Ser/Thr protein kinases [8]. Also within the 317 ELKs from *S. cellulosum* predominantly lysine residues were found in the catalytic loop [5]. However, dual-specificity was shown for an ELK with the catalytic loop sequence D-L-K-P-D-N [47] and therefore phosphorylation of tyrosine-residues can not be excluded without biochemical analyses for the myxobacterial ELKs. We therefore searched for predominantly serine/threonine specific PPs as well as predominantly tyrosine-specific PPs.

A significant expansion of PP2C-type PPs could be observed for *S. cellulosum*.

Experimental data indicate that the PP2c-type PPs can be involved in opposing ELKs in bacteria [48], [49], [50], [51], and indeed *S. cellulosum* harbors sixteen of these PP2c-type PPs, four times more than *M. xanthus*. Also several *S. cellulosum* genes, which encode PP2c-type PPs, are co-organized with ELK genes.

The expansion of the SpoIIE-like PPs and the related expansion of anti anti-sigma factors only seen in *S. cellulosum* is a significant differences to *M. xanthus*. Expansion of that group of PPs was so far only known from *S. coelicolor* and *S. avermitilis* [15]. Our phylogenetic analysis suggests that the SpoIIE-like PPs in *S. cellulosum* and *S. coelicolor* originated similarly. We interpret that as a reflection of their habitats and their life styles. Myxobacteria are as the Actinobacteria predominantly soil organisms, but in contrast to the bacteriolytic *M. xanthus*, the cellulolytic *S. cellulosum* as well as *S. coelicolor* can grow on various carbon sources, are very active secondary metabolite producers [4], [20], [21], and might therefore both prefer habitats with high cellulose-degrading activities.

The anti anti-sigma factors (COG1366) share a common domain, the STAS domain, with anion transport proteins [52] and a general role in NTP binding was suggested for that domain [53]. Without knowing the function of these STAS-domain proteins in *S. cellulosum* it is difficult to speculate why *M. xanthus* does not harbour similar number of these proteins.

Several PPPs could be identified in the myxobacterial genomes. We observed a slight expansion of these PPPs in the myxobacterial genomes and therefore do not rule out that they might also play a role in antagonizing ELKs. Nevertheless, we assume that these PPP-PPs are not necessarily all involved in myxobacterial developmental processes as we found in the genome of the non-fruiting *A. dehalogenans* only slightly less PPP numbers.

The finding, that the number of PTP-PPs is rather low in the myxobacterial genomes encoding high numbers of ELKs supports the hypothesis, that the myxobacterial ELKs represents predominantly Ser/Thr protein kinases.

As already mentioned, there is a significant lack of coorganization of protein kinase and protein phosphatase genes, especially in myxobacterial genomes. Analysing more than one hundred putative myxobacterial PPs we could identify only one direct kinase-phosphatase gene pair for *M. xanthus* (mxan7161–mxan7162), for *S. cellulosum* (sce2320–sce2321), and one for *A. dehalogenans* (adeh3699–adeh3700). However, based on the assumption that a gene distance of up to five genes might be significant fourteen and three additional pairs could be described for *S. cellulosum* and *M. xanthus*, respectively. This is still a very low number considering the existence of 317 and 99 ELKs in these organisms. Of course, it can not be ruled out that there is a significant degree of co-organization of kinase and substrate genes or phosphatase and substrate genes. We are far from knowing the phosphoproteome from myxobacteria but some proteins have been identified in *M. xanthus*. For example, the protein kinase gene *pktD1* (*pkn4*) forms an operon with the *pfk* gene, encoding the substrate phosphofructokinase [54]. The *pkn4* gene region is interesting as it represents the only ELK region where synteny among seven myxobacteria could be detected [5]. For *S. cellulosum* a phosphoproteome analysis was performed and 53 proteins were identified [7]. For 15 of them, a gene proximity to an ELK gene up to a distance of five genes was found. However, in a genome where approximately every thirtieth gene is an ELK gene, one must be careful not to over interpret gene proximities. The lack of coorganization on the gene level does not rule out a complex network of ELKs, PPs and corresponding phosphoprotein substrates. For *M. xanthus* a cascade of phosphorylation events involving activities of at least two ELKs leads to activation of the important regulator MrpC [8], [55].

The identification of putative PPs in *M. xanthus* and *S. cellulosum* was based on HMM searches using established profiles and assignments of proteins to COG groups. As we can not rule out that we missed only slightly altered non-HMM-conforming enzymes, Blastp searches were performed. When indicated, we added putative phosphatases identified by Blastp searches. However, the number of proteins we propose here should be regarded as the minimal complement of functional PPs. Regarding the high numbers of proteins for which neither a function nor a COG group could be defined (*M. xanthus* 2981; *S. cellulosum* 4132), it is still possible that further PPs with new catalytic mechanisms will be identified in the future just as it happened for the new family of PtpZ phosphatases. Based on the current numbers, the ELK/PP ratios seemed to be quite high for the multicellular myxobacteria compared to other prokaryotic and eukaryotic organisms. A possible explanation for the elevated ELK/PP ratios in *M. xanthus* and *S. cellulosum* could be that after commitments of cells for development protein phosphorylation events do occur which are not reversed by PPs any more (cells could pass beyond a reversible window of time into a “point of no return”).

However, for eukaryotic systems, it is speculated that the total number of protein phosphatase holoenzymes might even exceed the protein kinase repertoire because the specificity of many of these enzymes is in fact mediated by accessory proteins [56], [57]. If myxobacterial PP activity is also regulated by accessory proteins, the low number of PPs could still oppose the action of all these ELKs. Therefore, we need to understand how the myxobacterial PPs are working. Exploring the phosphoproteome of myxobacteria and its impact on regulating multicellular development is a challenging goal for the coming years.

## Materials and Methods

### Genomes used for comparison

Our genome comparisons are based on the COG database (3 Eukarya, 13 Archaea, 50 Bacteria) and in addition six myxobacterial and two actinobacterial genomes (Table S1). The following genome sequence files were used for comparisons: *M. xanthus* DK1622 (NC_008095); *S. cellulosum* Soce 56 (NC_010162); *A. dehalogenans* 2CP-C (NC_011891) (*A. dehalo*.), *A. dehalogenans* 2CP-1 (NC_011891), *A. sp.* K (NC_011145), *A. sp.* FW 109-5 (NC_009675), and *S. coelicolor* (NC_003888), *S. avermitilis* (NC_003155) For the COG database the following link was used: http://www.ncbi.nlm.nih.gov/COG/grace/wiew.cgi”. For COG and pfam searches the service of the integrated microbial genome homepage (http://img.jgi.doe.gov/cgi-bin/pub/main.cgi) was used.

### Bioinformatic tools

Proteins assigned to COG groups [25] were checked for pfam profiles using pfam search (http://pfam.sanger.ac.uk/) [58]. Sequences from similar species of one genus were restricted to one genus. For example in case of the four *Anaeromyxobacter* species we used only sequences from *A. dehalogenans* 2CP-C (*Adeh*). Multiple sequence alignments and bootstrap trees with 1000 replicates were generated using ClustalX2 [59] and the Neighbour-joining method [60]. Trees were visualized using the program TreeView X. [61]. Domain informations are given as pfam numbers and domain organizations were analyzed using the conserved domains database (CDD, http://www.ncbi.nlm.nih.gov/Structure/cdd/). Orthologs were defined as candidates with a significant BLASTP E-value (≤10^−10^) and having one candidate as the best-matching homolog of the other candidate in the corresponding organism by doing reciprocal Blastp searches [62]. When orthologs were identified, synteny was determined by manually investigating the gene neighbourhood of the putative PP genes. Co-organization of kinase and phosphatase genes was considered significant, when the genes were not more than five genes apart from each other.

## Supporting Information

Figure S1 Multiple ClustalX alignment of the myxobacterial PP2C-type PPs.
(3.20 MB PDF)

Figure S2 Multiple ClustalX alignment of the myxobacterial SpoIIE-like PPs.
(3.12 MB PDF)

Table S1 Complete numbers of certain COG proteins in 72 different eukaryotic, archaeal and bacterial genomes based on the 66 genome COG database and data obtained from the IMG portal.
(0.04 MB XLS)
