# Supplementary material for: The Phosphatomes of the Multicellular Myxobacteria Myxococcus xanthus and Sorangium cellulosum in Comparison with Other Prokaryotic Genomes
Source: PLoS One. 2010 Jun 17;5(6):e11164. doi: 10.1371/journal.pone.0011164 (PMC2887360; doi:10.1371/journal.pone.0011164)
Supplement: Figure S1 — Multiple ClustalX alignment of the myxobacterial PP2C-type PPs. (3.20 MB PDF) [file pone.0011164.s001.pdf]

Figure S1: multiple sequence alignment of myxobacterial proteins assigned to COG0631

```
Sce2351   1   ---------- ---------- ---------- ---------- ---------- ---------- ---------- ----------
Sce3569   1   ---------- ---------- ---------- ---------- ---------- ---------- ---------- ----------
Sce2040   1   MSDQTVRETP TEALEVEGLP GATSAGTDSS SHAGKALHDS PHGAASLESG KAGSVEAGKP VNWKPPPLKT RPAANAAQKA
Sce6171   1   ---------- ---------- ---------- ---------- ---------- ---------- ---------- ----------
Sce2784   1   ---------- ---------- ---------- ---------- ---------- ---------- ---------- ----------
Sce3096   1   ---------- ---------- ---------- ---------- ---------- ---------- ---------- ----------
Sce2855   1   ---------- ---------- ---------- ---------- ---------- ---------- ---------- ----------
Sce0638   1   ---------- ---------- ---------- ---------- ---------- ---------- ---------- ----------
Sce4754   1   ---------- ---------- ---------- ---------- ---------- ---------- ---------- ----------
Sce8596   1   ---------- ---------- ---------- ---------- ---------- ---------- ---------- ----------
Sce7899   1   ---------- ---------- ---------- ---------- ---------- ---------- ---------- ----------
Sce6950   1   ---------- ---------- ---------- ---------- ---------- ---------- ---------- ----------
Sce2514   1   ---------- ---------- ---------- ---------- ---------- ---------- ---------- ----------
Sce3126   1   ---------- ---------- ---------- ---------- ---------- ---------- ---------- ----------
Sce3795   1   ---------- ---------- ---------- ---------- ---------- ---------- ---------- ----------
Sce6484   1   ---------- ---------- ---------- ---------- ---------- ---------- ---------- ----------
Mxan4398  1   ---------- ---------- ---------- ---------- ---------- ---------- ---------- ----------
Mxan1412  1   ---------- ---------- ---------- ---------- ---------- ---------- ---------- ----------
Mxan5349  1   ---------- ---------- ---------- ---------- ---------- ---------- ---------- ----------
Mxan2044  1   ---------- ---------- ---------- ---------- ---------- ---------- ---------- ----------
Adeh3691  1   ---------- ---------- ---------- ---------- ---------- ---------- ---------- ----------
Adeh0733  1   ---------- ---------- ---------- ---------- ---------- ---------- ---------- ----------

Sce2351   1   ---------- ---------- ---------- ---------- ---------- MLRDVAFLAD MGGGSAFEVA GG--------
Sce3569   1   ---------- ---------- ---------- ---------- ---------- ---------- --VNPPLHIE AA--------
Sce2040   81  GIAVKPVKAP SASSPEKVGR KLPPPPRVRL TAGGSREEDA DGSAARSDDL GDAVTPDAPP AAAQAHASLA AAPYDGALDE
Sce6171   1   ---------- ---------- ---------- ---------- ---------- ---------- ------MRAI AS--------
Sce2784   1   ---------- ---------- ---------- ---------- ---------- ---------- -----MLRTR FA--------
Sce3096   1   ---------- ---------- ---------- ---------- ---------- ---------- ------MRAT AA--------
Sce2855   1   ---------- ---------- ---------- ---------- ---------- -------VSR ILPRFDYRIE FA--------
Sce0638   1   ---------- ---------- ---------- ---------- ---------- ---------- -MVRPTIAIE AG--------
Sce4754   1   ---------- ---------- ---------- ---------- ---------- ----MPVYRT SAVEHAAAVA VA--------
Sce8596   1   ---------- ---------- ---------- ---------- ---------- ---------- --VQRHLVFE AA--------
Sce7899   1   ---------- ---------- ---------- ---------- ---------- ---------- --VSQTFRIE VA--------
Sce6950   1   ---------- ---------- ---------- ---------- ---------- ---------- ------MRIQ SF--------
Sce2514   1   ---------- ---------- ---------- ---------- ---------- ------MMPP TESSPHLKIE FAQ-------
Sce3126   1   ---------- -VLQSTIKRA SIPSYGGDDD DDDDDDDGHE REEPSAVPII YDDEAAVDEP TSSSPLLLIS AV--------
Sce3795   1   ---------- ---------- ---------- ---------- ---------- -------MGE FRTSSRTRPV AA--------
Sce6484   1   ---------- ---------- ---------- ---------- ---------- -----MGLS- ------ENIR FY--------
Mxan4398  1   ---------- ---------- ---------- ---------- ---------- ----MFAVA- ------LTTE AF--------
Mxan1412  1   ---------- ---------- ---------- ---------- ---------- ---------- ------MRMD SA--------
Mxan5349  1   ---------- ---------- ---------- ---------- ---------- ----MSSTAG QTAASRLKII SA--------
Mxan2044  1   ---------- ---------- ---------- ---------- ---------- ---------- ------MRIE VA--------
Adeh3691  1   ---------- ---------- ---------- ---------- ---------- ---------- ------MRLS HA--------
Adeh0733  1   ---------- ---------- ---------- ---------- ---------- ----MAATP- -----VLTIA AH--------

Sce2351   22  ---------- ---------- ---------- ---------- ---------- ---TVTGTDH LVAGRPNQDA YAFRAEGGS-
Sce3569   10  ---------- ---------- ---------- ---------- ---------- -----GDSDV GHQREHNEDS FAVDLDLG--
Sce2040   161 TPAAEAAHGG AAGRFAGEAP GGGEPDEGGQ RPDGMDGAAT PPDEPRRPEV VLRLFGRTDV GQVREHNEDN FIVADLTKAS
Sce6171   6   ---------- ---------- ---------- ---------- ---------- -----GMTDV GLQRDHNEDS YAVLSEYD--
Sce2784   7   ---------- ---------- ---------- ---------- ---------- -----GMTDT GLLRDHNEDN LLVSEEYR--
Sce3096   6   ---------- ---------- ---------- ---------- ---------- -----GLSDV GRERVHNEDR YILLPEFN--
Sce2855   15  ---------- ---------- ---------- ---------- ---------- -----ASTDV GLVRPSNEDR ILCCPELA--
Sce0638   11  ---------- ---------- ---------- ---------- ---------- -----AEALV SRRRASNEER SGELPCLG--
Sce4754   18  ---------- ---------- ---------- ---------- ---------- -----ARTDV GLSREHNEDT FLISDLARGL
Sce8596   10  ---------- ---------- ---------- ---------- ---------- -----GISHP GMVRDTNEDS FAILEPRG--
Sce7899   10  ---------- ---------- ---------- ---------- ---------- -----GETNV GRKRNHNEDN FGIMVEYG--
Sce6950   6   ---------- ---------- ---------- ---------- ---------- -----GRTDV GRHRRANEDA FFRDDELR--
Sce2514   17  ---------- ---------- ---------- ---------- ---------- ----ASDAGR DPNKQVNEDA CGYAETKFG-
Sce3126   61  ---------- ---------- ---------- ---------- ---------- -----GQTDP GQKRKKNEDC YLCLDEHY--
Sce3795   15  ---------- ---------- ---------- ---------- ---------- -----AVTDV GRQRKHNEDN VLVKSDLG--
Sce6484   10  ---------- ---------- ---------- ---------- ---------- -----AATDV GRMRDHNEDN YLVDKKLA--
Mxan4398  11  ---------- ---------- ---------- ---------- ---------- -----GLTDV GRRRQHNEDA MLVDAALG--
Mxan1412  6   ---------- ---------- ---------- ---------- ---------- -----GQTHI GR-RPHNEDA FCVAPELG--
Mxan5349  18  ---------- ---------- ---------- ---------- ---------- -----GLTDV GRKRNHNEDS FLIDDELQ--
Mxan2044  6   ---------- ---------- ---------- ---------- ---------- -----GSTHV GMKRNHNEDN FLMLPEEF--
Adeh3691  6   ---------- ---------- ---------- ---------- ---------- -----GASDV GRKRAHNEDA FLLLPEEQ--
Adeh0733  12  ---------- ---------- ---------- ---------- ---------- -----GVTDV GRRRDHNEDA FLVDPALG--

Sce2351   48  ---------- ------LVAV VCDGCGSGAR SEVGAALGAR IVAEQVLGAL RRGGDLDSPA TWEEVRRAAL AP------LR
Sce3569   33  ---------- -------LFV VADGIGGAAG GEVASRMAVD IVRSFCKDGE GEITQPTGYD EEVTP-LELR --------LV
Sce2040   241 RGLMEMDRYQ VVGERGALLG VCDGMGGAAA GEVASQLAVD IIYQRMS--- ------AAGP PENHDELAAR --------LV
Sce6171   29  ---------- -------LFI VADGMGGHRA GDVASKLATE SIADFFRSTS REDATWPFHF DTS-LSEEEN R-------LQ
Sce2784   30  ---------- -------VVA VADGMGGHRS GDVASQLAVS TLSDFLSVTV GRDATWPFPA DPN-LTEEEN Y-------VV
Sce3096   29  ---------- -------VYV VADGMGGHEA GEVASRIAAS AIAGYFREAN GKRRK----- -----ASTGD R-------LR
```

Figure S1: multiple sequence alignment of myxobacterial proteins assigned to COG0631

```
Sce2784    30  ---------- -------VVA VADGMGGHRS GDVASQLAVS TLSDFLSVTV GRDATWPFPA DPN-LTEEEN Y-------VV
Sce3096    29  ---------- -------VYV VADGMGGHEA GEVASRIAAS AIAGYFREAN GKRRK----- -----ASTGD R-------LR
Sce2855    38  ---------- -------LFG IADGMGGHAA GEVAAQIAVD TVRGEVLRPP AAAILDAYVA DPNIEARREV FA-----LLR
Sce0638    34  ---------- -------LFL VT----GRPG GEVVSRTAIE MVHPAVDAAS VDDLGAGEMI APLCRDEVR- ----------
Sce4754    44  AHGPAAAQAS VEPPLALSLA VYDGSGGASS PDAASRLAAR IVHATLT--- ------RSAP -RSSDALERS --------LL
Sce8596    33  ---------- -------LFL LADGMGGRAA GEVASRMAID TVRDFFDDPD STWPVAVGVG PPTTRGSEAR YVDQGLPLLV
Sce7899    33  ---------- -------LFM VADGMGGHAS GEVASKMAVD AMQDFFAQTQ DDPERTWPYK MDRSKGYEEN R-------LI
Sce6950    29  ---------- -------LYI VADGMGGHAA GEVASAEAVE TIYGMVKRG- LPAIGDPN-E PLTQPKARAV LR-----ILE
Sce2514    42  ---------- ------YLCV LCDGMGGHYG GSEASRTAIQ TIFEMIERTP ATVDPSGA-- ---------- --------LK
Sce3126    84  ---------- -------LFA VADGMGGHAG GDVASRLAVD TIARAFKDQS FVDSDAESPY PDV--PRRGS E-------LA
Sce3795    38  ---------- -------LFV VADGMGGHNA GNVASALATK SLDNFFEATR AGSLPGPVPA DEQELDPEAR R-------VV
Sce6484    33  ---------- -------LFV VADGMGGHAA GEVASALAVR IIHEELKKE- RDLIENQARS NVRRAAMKEV LS-----LLE
Mxan4398   34  ---------- -------LYV VADGMGGHSA GEVASNRATE VVKQHISAN- RHLLKDLG-I NPTPDSRSAA AA-----LVE
Mxan1412   28  ---------- -------LFV VADGLGGQEG GEVASRCVVD TFVGFGLRLG QDRDSTWPTV PDPRRSREEN ---------L
Mxan5349   41  ---------- -------LYV VADGMGGHAG GGTASRIAVE TIDKEMRRA- REGKDNPF-L SVPNLQDSPI PE-----ALR
Mxan2044   29  ---------- -------LFC VADGMGGHSS GEIASRIAVD ELGEFYKLTS KDQDCTWPFK MDKTRNYDEN R-------LA
Adeh3691   29  ---------- -------LYC VADGMGGHAS GEVAARIAVE EMAEFFRITG RDDEATWPFR PDPARDADEN R-------LL
Adeh0733   35  ---------- -------LYV VADGMGGHAG GGTASRLAVE TIQESVRAA- RDGSPEVF-E GANGVEDSRL PD-----LLR

Sce2351   107  DVAAGMGGSF TEVLSTFFLF TVVGLAISGD RACAFSLGDG LIALGDELLR LGPFPRDEPP YLAYGLLD-R PPGGEAPRFT
Sce3569    88  SSIWRANERI FEVGQRDPLC YGMGTTFAGV YVDGSS--AC IAHVGDSRVY RYRKRRLERL TEDHSLWN-E HLNTGEQLPD
Sce2040   304  QSIEAAGLRI FSEAKLDRTR RGMGTTSTIA ALMDDH--LF LGQVGDSRAY VLRGDRLVQV TRDQSLVN-Q LIEAGQLTEE
Sce6171    85  AGIRVANRQI FERSIRSRDC AGMGTTVVGA LFSKKKNRIY VGHVGDSRAY RVRKGSISQL TRDHSLFN-D YIMAMPELTE
Sce2784    86  TGLRLANRRI FDRSLKTLAD FGMGTTIVAA MFSKDAEHVT VGHVGDSRCY RLRDSQITQL TRDHSLVS-D AAHMAPWMTE
Sce3096    76  AAVTYANARI FAHADDSRFY RGMGTTVVAA AFSPRERKFY VAHAGDSRCY LLRNGDISQL TRDHSLIA-D ALLERPDLTE
Sce2855    97  RVGEAAHAAI LEARRTEPSY QGMGTTLDFV VLAHSR--AF FAHAGDSRAY LVRPTTTVQL TQDHALYD-T LRAAGNPTPA
Sce0638    83  LMMSPRRTGR SEDERPGPGP SGVQVTFAGV LLAPGT--AY IAYAGELHIY RFRGGKLEAR TREDAALDGG ALDRSSSPDA
Sce4754   106  EAIGEAGRAV RDNARRSSQH RNSGTTATVA ALCDER--LL LAQVGDSRAY LLRSGVLTQL TRDQTLLQ-S LLDSGKLRPE
Sce8596    97  AGIQLANGRI FSAARRDREK RGMGTTIAAV LARDGF--IA VAHVGDSRVY RLRDHRLEQL TRDHSLVN-E CIRLGHLLPE
Sce7899    90  TGIKLANLRI YETAQREAKK RGMGTTFVGI FTANDG--VY VAHVGDSRVY RFREGRLEML TEDHSLLN-D YIKMKRLTPE
Sce6950    86  GAIQAATYMV FAIAELDRDK SGMGTTISAA LIVGGA--LV TGQVGDSRIY QVRNGTAVQM TEDHTLIA-W QIKNGLISVD
Sce2514    87  AAIEEAGRRV YQLGGPPENR VRPGSTVVAM VLHERG--VD VAHVGDSRAY VIRSHQIYPL TRDHSMVQ-G MIDAGMLTEA
Sce3126   139  VAIQQANQAI YERAHAERSL TGMGTTLVSA RFSPNKQRLY IGHVGDSRCY RLRDNELTQL TTDHTMGA-A GITG-P----
Sce3795    95  AAIRKANHDV FVISNTYTQH QGMGSTVVAA YVSCETEQIH VGHVGDSRCY RIRHGEIEQL TKDHSLIN-D ALALKPDLSQ
Sce6484    91  HAVQRACARI HEEAKADTTK RGMGTTLSAL LIAGSH--GY IAHVGDSRIY LLREGRIQQV TEDHTVYN-E LIKRGKLTRD
Mxan4398   91  VSVQRACADI YRTAMSDATK RGMGTTFVCL AVGGNK--GV IGHVGDSRVY LVRHGQCHRL TEDHTLVA-A QLKAGTITKD
Mxan1412   83  LAACSALAQR NLQAQRVGRL REMASTVVAL AVSEHG--AA VAHVGDSRLY RLRGGKLESL TRDHSLIE-E LRDAGMEPPG
Mxan5349   98  TAVERACLAI FTAAQEDARL SGMGTTVISL VVRDEH--AF FAHVGDSRAY LIRGDLIQQI SEDHSLVN-E QIKAGMITPE
Mxan2044   86  TGVKLANARI FEKACSESKY KGMGTTIVTV HFSQS--AVY VGHVGDSRVY YFRGGALKQV TEDHSLLN-D YLKAKKLSPE
Adeh3691   86  TGVKLANLRI HERARADERL HGMGTTLVAA QFPRDGREVL VGHVGDSRAY LFRRGALRQL TEDHSLLN-D FRRTRQLTPA
Adeh0733   92  EAVEAACARI FQTAQGDPGL AGMGTTVTAA LIDGRV--AF VAHVGDSRCY LLREGRIYQV SEDHSLVN-E QLKAGTISAD

Sce2351   186  VHRAFPSSAL RTALLGTDGA IDLLESSSRQ IPRGGEVGPL SRFWEDDRYF QNPDAVRRRL ALIN---RSV TRPVWKEERM
Sce3569   165  DVDPLLARNV VTRALGMGKT VDVATRVE-- RPEAGDL--L LICSDGLSGP VPELEIAELL AQES---DLA ATARSLIDRA
Sce2040   381  EAETFEHNNI ILQALGTADS VQVDLTFV-- ELKRGDT--L MLCSDGLSGM VRNEEIREVL RSVD---DPI EACKVLTDRA
Sce6171   164  EQRAELPRNV ITRALGMHDS VSVDLISD-- EPQPGDV--Y LLCSDGLSGM LSDDQILHIV SSTE---EVP EMCRRLIAKA
Sce2784   165  EEVRQLPANV ITRALGIRED VLVDLVTD-- ETRHGDV--Y LLCSDGLSGL VSDDQILELV MSSG---SLD AACKALIDRA
Sce3096   155  RDLAYLPRNV ITRALGIGPS VDIDLRAE-- RVEAGDV--F LLCSDGLHGL VDDREIARIV EDNA---VLA DACGKLIDCA
Sce2855   174  RK---PQRNP LVNAIGLAGT VAVDTVFV-- DLSRGDR--I VLCTDGVHNA IESEASLSRF CSKG---EPE QVAAGLLGHA
Sce0638   161  PAVLGQHASA ATRALGCDAA VEMTAQVE-- DTEPGDI--F LACSGGLWGS VSEQRIAGIL AAHR---ELR LAASLLMDCA
Sce4754   183  EAATFEHRGV MLQALGLREH LRIALSAV-- ELRREDT--L LLCTDGLSDL IDDQHIAAAL DAHP---DPA AAASALIDAA
Sce8596   174  QASSFPLQHV ITRALGTDET VDVETRID-- AIEGGDV--L LLCSDGLSGV VPAQDIASIL ATHT---DLA GIASRLVKRA
Sce7899   167  EIANFPHKNV IVRALGMKDT VKVDTRFE-- VPQLNDT--Y VLCSDGLSGP VSDLEMTQIL TEHQ---DIQ VATAKMIERA
Sce6950   163  EAKSSPHRNV ITRAVGNRDY VEVDTSIV-- AVEEGDR--Y LLCSDGLHGY LHA-EEEVAS IASK---GGD LAVDLFIDLA
Sce2514   164  QAIGHPDANK ITRALGMRPE VEVEVRPEPM ELYPGDV--L IQSSDGLTDL VLPGDILGCT RQALASGALD HACRMLVQLA
Sce3126   212  ------MANH LSRAIGISPA VKVDLIIA-- RPHPGDV--Y LLCSDGLSKM TTHEAIRDIL LAEP---DPE KASQALIEKA
Sce3795   174  DELARLPKNI ITRALGMKDA VKVDIRSE-- HTQPGDV--F LLCSDGLSGM ISEQQMLDVF DITQ---DPH EACELLIEMA
Sce6484   168  QIEKVAQKNA ITRAVGVYER VEVDTLTI-- EVLPGDQ--F LLASDGLHGY IAHTAELEPF FEEE---NGE IAANGLIDLA
Mxan4398  168  QAANSQYRNV ITRAVGIQES VQVDTLIV-- DLVPGDM--F ILCSDGLHGY VED-EEVLPL IAGI---APA DLPKRFIDVA
Mxan1412  160  GSGN--LRHL ITRALG-TEN AEPTVQRL-- QTEPGDV--F LLCSDGLYEP LGVEGLMKRL TMSS----AR EVCDALVADA
Mxan5349  175  EAKHSRYKNI ITRSVGFEEE VQVDVMGL-- VSEPGDV--F LLCSDGLANM MEDREIHETV VKAR---TFE EVPKRLIDFA
Mxan2044  163  EIENFPHKNV IVRALGMKEN VQVDVSRV-- EPQEDDV--F LLCSDGLSGM VTDAQMQEIL QRTP---ELE KACSQLIDMA
Adeh3691  165  EIEAFPHKNV IVRALGMKES VEVDLLRE-- PLEDGDV--V LLCSDGLSGM VPDARIAEVL RAVPG--DLR RAAQALVDAA
Adeh0733  169  EAKHSRFKNI ITRSVGFEAQ VQVDLMGI-- ELEGGDA--L VICCDGLSNL VDDDEILQ-I VDEC---PID EAPARLVALA

Sce2351   263  EREGGLLRDD TTVVVVRRAQ GARD------ ---------- ---------- ---------- ---------- ----------
Sce3569   238  NQHGG--PDN VTCVLVRWNA ---------- ---------- ---------- ---------- ---------G G---------
Sce2040   454  NQAGG--HDN ITVVVAKFDG DGLALPELED IEDLRYQKYA LPEHLLAQNA AASEPARKVK ELDEKKISQR PPSPKSWLSG
Sce6171   237  NENGG--EDN ITALVIRID- EQDDA----- ---------- ---------- ELPNSATIGP PADSQQVSMG DRSTVPAVSA
Sce2784   238  NYFGG--TDN ITVVLARVE- EVPDG----- ---------- ---------- KRP------- --------FN DEPTQ----E
Sce3096   228  NENGG--RDN ITAVLIRIEE TASAS----- ---------- ---------- WSQRQG---- --------VK SRSAR-----
Sce2855   244  RERGG--HDN ASVIVIDIKE RFVKRADDAG PSSRDLSVLS ACPLLAGMSP AAVLGALAAG VEVELGAGDR IPRDVASDLV
Sce0638   234  HENGE--PEQ AMCVLARVAG SVG------- ---------- ---------- ---------- ---------- ----------
Sce4754   256  LAAGG--RDN VTALVTRFDG PWLAPP---- ---------- ---------- -ASDRATEIV EIAR--FSEG P-TRRS----
Sce8596   247  NENGG--PDN VTCVVVRWRA S--------- ---------- ---------- ---------- --------PG GPTRR-----
```

```
Sce4754   256 LAAGG--RDN VTALVTRFDG PWLAPP---- ---------- ---------- -ASDRATEIV EIAR--FSEG P-TRRS----
Sce8596   247 NENGG--PDN VTCVVVRWRA S--------- ---------- ---------- ---------- --------PG GPTRR-----
Sce7899   240 NENGG--PDN VTCVLVRWTQ ---------- ---------- ---------- ---------- ---------- ----------
Sce6950   235 NRRGG--RDN ITAVLVEVG- ---------- ---------- ---------- ---------- ---------- ----------
Sce2514   242 NHRGG--HDN ITVQMVRVLE TGSRSLTIAH GPPGAAGPGD GVNPARASRP QETVAMTRSD AAAIGQLHAT STPLPHGHGA
Sce3126   280 NAGGG--RDN ITVILVRVSD PKDLA----- ---------- ---------- RLASRAG--- --------AS AGASAG----
Sce3795   247 NEAGG--TDN ISALIVRIQL DAQDE----- ---------- ---------- RQPNSAELDD TRPLHVDEFG ETVPPAAVHE
Sce6484   241 NRKGG--KDN ITAILVRLGQ GDHRDSVRAR LLALKRDVLA KMPLFARLQE REMLRVMQVA EVLSFEPGQI VVREGDRGDE
Mxan4398  240 NERGG--KDN ITAVVVKVAG EGSSVSEESS EAQSRMEALR KIPLFRHLTY KEQTAVLSIA TTRTYPAGRE IVVEGQPGEE
Mxan1412  229 YEAGG--KDN ITAVVLRVAE A--------- ---------- ---------- ---------- ---------- ----------
Mxan5349  248 NERGG--DDN ITVIVVRVEA ---------- ---------- ---------- ---------- ---------- ----------
Mxan2044  236 NAAGG--NDN VTCVLARYHA A--------- ---------- ---------- ---------- ---------- ----------
Adeh3691  239 NSAGG--ADN VTCVLVQAQG ---------- ---------- ---------- ---------- ---------- ----------
Adeh0733  241 NDRGG--DDN ITVIVIRAGL APVMVG---- ---------- ---------- ---------- ---------- ----------

Sce2351   286 ---------- ---------- ---------- ---------- ---------- ---------- ---------- ----------
Sce3569   257 ---------- ---------- ---------- ---------- ---------- ---------- ---------- ----------
Sce2040   532 ADSDMDDDGD DYDHVASPLP ADDALDERNA RAPVQGDEPI MIPTDGAPQW LVILMIVSAV ACVTIAGYYL LR--------
Sce6171   289 SRSEKS---- ---------- ---------- ---------- ---------- ---------- ---------- ----------
Sce2784   271 SR-------- ---------- ---------- ---------- ---------- ---------- ---------- ----------
Sce3096   263 ---------- ---------- ---------- ---------- ---------- ---------- ---------- ----------
Sce2855   322 AYLVLDGAVN LPDGRR---- VGSSGLLFPE SLVGARRKGD LPVAAARTRL IRIRKDDFAE VCEHDAELAA ALYQRLARYL
Sce0638   254 ---------- ---------- ---------- ---------- ---------- ---------- ---------- ----------
Sce4754   301 ---------- ---------- ---------- ---------- ---------- ---------- ---------- ----------
Sce8596   272 ---------- ---------- ---------- ---------- ---------- ---------- ---------- ----------
Sce7899   257 ---------- ---------- ---------- ---------- ---------- ---------- ---------- ----------
Sce6950   251 ---------- ---------- ---------- ---------- ---------- ---------- ---------- ----------
Sce2514   320 PAAASGAAAG MAAAGDAPAP YPASINNAIT VVPTGSPDTP RPASTSDAVT VVPAGSTGAP GLRPASPRSA ATAASAGSEN
Sce3126   317 ---------- ---------- ---------- ---------- ---------- ---------- ---------- ----------
Sce3795   300 GQLPVDEVDL DIAAQQVHSI ADSGLRSQAH SAPGAVARMA AAPFTDRRSR AAAGSGRYAA VTTAARPPVK EAPPSKPAKR
Sce6484   319 LFIVLSGLVR IIRGESVLSE VGPGEHFGEM ALIRSMPRSA TVSAVEQSEL IAVRRADFFE ILRKEHELAV KLLWQFLGVL
Mxan4398  318 LFVVIRGRVA IEKNGVEIAE LRAGGHFGEM GLIDNAPRSA TVRATEPTRT MVISRPDLMG LMKRESILAV KMLWSFVQVL
Mxan1412  247 ---------- ---------- ---------- ---------- ---------- ---------- ---------- ----------
Mxan5349  265 ---------- ---------- ---------- ---------- ---------- ---------- ---------- ----------
Mxan2044  254 ---------- ---------- ---------- ---------- ---------- ---------- ---------- ----------
Adeh3691  256 ---------- ---------- ---------- ---------- ---------- ---------- ---------- ----------
Adeh0733  264 ---------- ---------- ---------- ---------- ---------- ---------- ---------- ----------

Sce2351   286 ---------- ---------- ---------- ---------- ---------- ---------- ---------- ----------
Sce3569   257 ---------- ---------- ---------- ---------- ---------- ---------- ---------- ----------
Sce2040   603 ---------- ---------- ---------- ---------- ---------- ---------- ---------- ----------
Sce6171   294 ---------- ---------- ---------- ---------- ---------- ---------- ---------- ----------
Sce2784   272 ---------- ---------- ---------- ---------- ---------- ---------- ---------- ----------
Sce3096   263 ---------- ---------- ---------- ---------- ---------- ---------- ---------- ----------
Sce2855   398 ALGTG----- ---------- ---------- ---------- ---------- ---------- ---------- ----------
Sce0638   254 ---------- ---------- ---------- ---------- ---------- ---------- ---------- ----------
Sce4754   301 ---------- ---------- ---------- ---------- ---------- ---------- ---------- ----------
Sce8596   272 ---------- ---------- ---------- ---------- ---------- ---------- ---------- ----------
Sce7899   257 ---------- ---------- ---------- ---------- ---------- ---------- ---------- ----------
Sce6950   251 ---------- ---------- ---------- ---------- ---------- ---------- ---------- ----------
Sce2514   400 VPAVAPTATD NAAVAPTTTD NVPAVAPGAS HGAPAVSPTT VDSAPAVSPT ATDELPPVAP AAAPSAKAVS PTAADGGSAM
Sce3126   317 ---------- ---------- ---------- ---------- ---------- ---------- ---------- ----------
Sce3795   380 ATEVRVARCA HCKHELFIGN RFCVECGAPI KP-------- ---------- ---------- ---------- ----------
Sce6484   399 ADRLDQTSRD LSTAREELAA EDITSEIFPD SEEGKSPFAP EERSSAR--- ---------- ---------- ----------
Mxan4398  398 SDRLRATNSE LSEARQELAV AQAI------ -----APFAE E--------- ---------- ---------- ----------
Mxan1412  247 ---------- ---------- ---------- ---------- ---------- ---------- ---------- ----------
Mxan5349  265 ---------- ---------- ---------- ---------- ---------- ---------- ---------- ----------
Mxan2044  254 ---------- ---------- ---------- ---------- ---------- ---------- ---------- ----------
Adeh3691  256 ---------- ---------- ---------- ---------- ---------- ---------- ---------- ----------
Adeh0733  264 ---------- ---------- ---------- ---------- ---------- ---------- ---------- ----------

Sce2351   286 ---------- ---------- ---------- ---------- ---------- ---------- ---------- ----------
Sce3569   257 ---------- ---------- ---------- ---------- ---------- ---------- ---------- ----------
Sce2040   603 ---------- ---------- ---------- ---------- ---------- ---------- ---------- ----------
Sce6171   294 ---------- ---------- ---------- ---------- ---------- ---------- ---------- ----------
Sce2784   272 ---------- ---------- ---------- ---------- ---------- ---------- ---------- ----------
Sce3096   263 ---------- ---------- ---------- ---------- ---------- ---------- ---------- ----------
Sce2855   402 ---------- ---------- ---------- ---------- ---------- ---------- ---------- ----------
Sce0638   254 ---------- ---------- ---------- ---------- ---------- ---------- ---------- ----------
Sce4754   301 ---------- ---------- ---------- ---------- ---------- ---------- ---------- ----------
Sce8596   272 ---------- ---------- ---------- ---------- ---------- ---------- ---------- ----------
Sce7899   257 ---------- ---------- ---------- ---------- ---------- ---------- ---------- ----------
Sce6950   251 ---------- ---------- ---------- ---------- ---------- ---------- ---------- ----------
Sce2514   480 TPTPMDSALA LPPTVPGAHP PVAYPNAPDA PAVLSPLPLS APAPPPMGRA AAPQPGSFGA PPHPGAAYGA PPHPAVPYGA
Sce3126   317 ---------- ---------- ---------- ---------- ---------- ---------- ---------- ----------
```

Figure S1: multiple sequence alignment of myxobacterial proteins assigned to COG0631

```
Sce2514   480 TPTPMDSALA LPPTVPGAHP PVAYPNAPDA PAVLSPLPLS APAPPPMGRA AAPQPGSFGA PPHPGAAYGA PPHPAVPYGA
Sce3126   317 ---------- ---------- ---------- ---------- ---------- ---------- ---------- ----------
Sce3795   411 ---------- ---------- ---------- ---------- ---------- ---------- ---------- ----------
Sce6484   445 ---------- ---------- ---------- ---------- ---------- ---------- ---------- ----------
Mxan4398  427 ---------- ---------- ---------- ---------- ---------- ---------- ---------- ----------
Mxan1412  247 ---------- ---------- ---------- ---------- ---------- ---------- ---------- ----------
Mxan5349  265 ---------- ---------- ---------- ---------- ---------- ---------- ---------- ----------
Mxan2044  254 ---------- ---------- ---------- ---------- ---------- ---------- ---------- ----------
Adeh3691  256 ---------- ---------- ---------- ---------- ---------- ---------- ---------- ----------
Adeh0733  264 ---------- ---------- ---------- ---------- ---------- ---------- ---------- ----------

Sce2351   286 ---------- ---------- ---------- ---------- ---------- ---------- ---------- ----------
Sce3569   257 ---------- ---------- ---------- ---------- ---------- ---------- ---------- ----------
Sce2040   603 ---------- ---------- ---------- ---------- ---------- ---------- ---------- ----------
Sce6171   294 ---------- ---------- ---------- ---------- ---------- ---------- ---------- ----------
Sce2784   272 ---------- ---------- ---------- ---------- ---------- ---------- ---------- ----------
Sce3096   263 ---------- ---------- ---------- ---------- ---------- ---------- ---------- ----------
Sce2855   402 ---------- ---------- ---------- ---------- ---------- ---------- ---------- ----------
Sce0638   254 ---------- ---------- ---------- ---------- ---------- ---------- ---------- ----------
Sce4754   301 ---------- ---------- ---------- ---------- ---------- ---------- ---------- ----------
Sce8596   272 ---------- ---------- ---------- ---------- ---------- ---------- ---------- ----------
Sce7899   257 ---------- ---------- ---------- ---------- ---------- ---------- ---------- ----------
Sce6950   251 ---------- ---------- ---------- ---------- ---------- ---------- ---------- ----------
Sce2514   560 PPPPGAAYGA PPPPGVPYGT PPPPGAAYGA SPHVGAAYGA PLHATEQDVP PPSVAFPGHN PAAPGGPPLA AAPPHHIAPA
Sce3126   317 ---------- ---------- ---------- ---------- ---------- ---------- ---------- ----------
Sce3795   411 ---------- ---------- ---------- ---------- ---------- ---------- ---------- ----------
Sce6484   445 ---------- ---------- ---------- ---------- ---------- ---------- ---------- ----------
Mxan4398  427 ---------- ---------- ---------- ---------- ---------- ---------- ---------- ----------
Mxan1412  247 ---------- ---------- ---------- ---------- ---------- ---------- ---------- ----------
Mxan5349  265 ---------- ---------- ---------- ---------- ---------- ---------- ---------- ----------
Mxan2044  254 ---------- ---------- ---------- ---------- ---------- ---------- ---------- ----------
Adeh3691  256 ---------- ---------- ---------- ---------- ---------- ---------- ---------- ----------
Adeh0733  264 ---------- ---------- ---------- ---------- ---------- ---------- ---------- ----------

Sce2351   286 ---------- ---------- ---------- ---------- ---------- ---------- --------
Sce3569   257 ---------- ---------- ---------- ---------- ---------- ---------- --------
Sce2040   603 ---------- ---------- ---------- ---------- ---------- ---------- --------
Sce6171   294 ---------- ---------- ---------- ---------- ---------- ---------- --------
Sce2784   272 ---------- ---------- ---------- ---------- ---------- ---------- --------
Sce3096   263 ---------- ---------- ---------- ---------- ---------- ---------- --------
Sce2855   402 ---------- ---------- ---------- ---------- ---------- ---------- --------
Sce0638   254 ---------- ---------- ---------- ---------- ---------- ---------- --------
Sce4754   301 ---------- ---------- ---------- ---------- ---------- ---------- --------
Sce8596   272 ---------- ---------- ---------- ---------- ---------- ---------- --------
Sce7899   257 ---------- ---------- ---------- ---------- ---------- ---------- --------
Sce6950   251 ---------- ---------- ---------- ---------- ---------- ---------- --------
Sce2514   640 PLHAHAYAAT GPRPAPAPSF SGPVSSSGAS SVPSTVRAPR GVYIALIASA SAVLLVLFVF LLWKLLGS
Sce3126   317 ---------- ---------- ---------- ---------- ---------- ---------- --------
Sce3795   411 ---------- ---------- ---------- ---------- ---------- ---------- --------
Sce6484   445 ---------- ---------- ---------- ---------- ---------- ---------- --------
Mxan4398  427 ---------- ---------- ---------- ---------- ---------- ---------- --------
Mxan1412  247 ---------- ---------- ---------- ---------- ---------- ---------- --------
Mxan5349  265 ---------- ---------- ---------- ---------- ---------- ---------- --------
Mxan2044  254 ---------- ---------- ---------- ---------- ---------- ---------- --------
Adeh3691  256 ---------- ---------- ---------- ---------- ---------- ---------- --------
Adeh0733  264 ---------- ---------- ---------- ---------- ---------- ---------- --------
```
