# Supplementary material for: The Phosphatomes of the Multicellular Myxobacteria Myxococcus xanthus and Sorangium cellulosum in Comparison with Other Prokaryotic Genomes
Source: PLoS One. 2010 Jun 17;5(6):e11164. doi: 10.1371/journal.pone.0011164 (PMC2887360; doi:10.1371/journal.pone.0011164)
Supplement: Figure S2 — Multiple ClustalX alignment of the myxobacterial SpoIIE-like PPs. (3.12 MB PDF) [file pone.0011164.s002.pdf]

Figure S2: multiple sequence alignment of myxobacterial proteins assigned to COG2208

```
mxan4562  1   ---------- ---------- ---------- ----MSSTNT RLKSAPASDE FPDDAALPPS ERTGTQEVTS ARESTATRLT
sce1779   1   ---------- ---------- ---------- ---------- MHSRGFLQKQ SNLDAIRAIV KERQAWNTLL REEGNFTDFM
sce2617   1   ---------- ---------- ---------- ---MHDREPE AMTRKRRTLA FLIDNVVGDY QSELRAGIER GAAAHDVNLL
sce3610   1   ---------- ---------- ---------- ---------- ---------- ---------- ---------- ----------
sce3741   1   ---------- ---------- ---------- ---------- ---------- ---------- ---------- ----------
sce5198   1   ---------- ---------- ---------- ----MTGEPQ DKMKKRRTIA LVMDYVRGEY QSALRIGVER AAEAHDVNLV
sce5201   1   MQGGVPHRRE AGLATAEPDG HRAHVSGARL HRRYLTREPR EMSEKRRTIA FLMDYVGGDY QSEVRFGIER AAEAHDVNLV
sce5204   1   ---------- ---------- ---------- ----MKRGPQ DTMKKRRTIA LLMDYVRGEY QSEVRFGVER AAEAHDVNLV
sce6543   1   ---------- ---------- ---------- ----MRAGTP SLIDASSLIL ILDVSRKLAA PCDLTELLEL IIKTGREVIG
sce8427   1   ---------- ---------- ---------- ------MAEI DPLLARQLRR LGLEDLDAPP DKETWHKVVA RISEHYRHVA
sce9114   1   ---------- ---------- ---------- ---------- ---------- ---------- ---------- ----------
adeh0917  1   ---------- ---------- ---------- ----MFAFVA QRTRTSLRLR LLVATVALLA LALAAAAMAF ERVARSVVVD
sce0876   1   ---------- ---------- ---------- --MSGRRFGT HRRRSMSTPP PMVGPGRTMS PADDSLGPAP SSSDWKGHLE
sce1777   1   ---------- ---------- ---------- ----MTSPAD ETRKELRRRG FFFGLKKFNT KFMLVTGFSV LLGALLNVLV
sce2321   1   ---------- ---------- ---------- ---------- -MAAKRHTIA LLTDYLCGEY QSEARFGVER AAEAHDVNLI

mxan4562  47  GPLGLAEGGT RTLIAPLEAG ELPNVAQIKG LRLDQILLLT TGVLVVLIVG LLAAL-SVAS TKSQFEETAL VSKERIQEQA
sce1779   41  ERCSALLEYV DKLKLDFDDL SLVHEAILEH ATSIENELEG YNRAIDDDLA VARQV-QRAL LPEASGAIAS SFDMAIFHKQ
sce2617   48  TAFSDPAMAR RPEVADRGTF YHLIGPETAD GVIVASSTLS PYGGMEVMRS FCRSF-APLP VCSIGTAIDG VPS-LMVDNA
sce3610   1   ---------- ---------- ---------- ---------- ---------- MTVAI-GTAC CVAAGEEIAG DEV-VVVR--
sce3741   1   ---------- ---------- ---------- ---------- ---------- MKLSV-EYAI LPKDGELVSG DAA-FVRA--
sce5198   47  IAVGETLSAH GPSEAAQNSI YRLVSAEAVD GIVVASATLC NFVGAEGVQR FCLDY-APMP LCSIGIPLEG IPS-IVVDNG
sce5201   81  IAFGETLALP GEGGAAQNSV YQLIGPETVD GIIVASATLC HHIGIEGMRE FFRAY-APLP VFSIGMAIDG IPS-LIVNNG
sce5204   47  IAFGETLALP GAASTAQNSM YHLIGAETVD GIIVASTTLC HHVGTEGMKR FCRSY-APLP ICSLGMAIDG IPS-LVVDNG
sce6543   47  ADRGSVFLYD EEAKELYSRV ATGEAQIRVS IDKGIAGECA RKRQTILVDD CYSDP-RFNQ EIDRRTGYHT KSLITVPLIG
sce8427   45  DDRSLLTRSL DLSTNEMGAL HRQLLAERDR --LRSVVVAI GDALTIFHDA ASSQV-EPSR PIEVGSTIST AKR-RFASKL
sce9114   1   ---------- ---------- ---------- ---------- ---------- -MRRC-GARG PHSHGGDIRG MGS-SSIGGC
adeh0917  47  AVHSHLAARA KEVHEAVVRF QGERALTVRN WAEAEAMQMS LDTGDPKFAE DYLRR-TIQD QGGAIGAAAL LAPDGEIRAA
sce0876   49  IRLGDLPGTR RPLTESHIVI GRAPGVQLTL DHYTVSRRHA ELFCDPFGRW WIRDLGSTNG TLVNDERINE KVLKPGDRIG
sce1777   47  ARQGLHELSQ KSAREIEGGL NEANREYLTN HLGDTAQQVN LKLGHAYADL RMLAA-IMQT MVDQGDQLDE LHKKVASTDF
sce2321   40  VAFGETRARQ GYTLDARGGI YSLIGATTVD GAVIMSSALT NHIGPEGAHE FCRSL-ASLP VCSVGLALDG VPS-VIVDNA

mxan4562  126 RELGQTVGQT IALTSATN-- ---------L RDNNYAFLEE VAGSIVKT-- --NPNILRVQ IFDPDGVRMA DSEGASEDKE
sce1779   120 LAQVGGDYYD FFYLPGER-- -------HAI GVYDISGHGV SSALIMAF-- --LKAQFAHA TKRLDSPGAI VD---WVNRS
sce2617   126 LGSEISVGHM ADEHGCRR-- ---------I AYIGGPSSSE EAALRADG-- --YRRALAAR ALPCDERLLV FG-GFTIPTG
sce3610   26  ---------- --QSPWVL-- ---------I GLADGLGHGP HAAQAASA-- --FCRYVEDH AS-MPIEDLL TG-AGEQLAS
sce3741   26  ---------- --GEDGVL-- ---------F AVIDALGHGE HAAAAANV-- --ALKALADA PSGEDVRALI DR-LHARLRG
sce5198   125 LGIEMAAGHL LDDHACRR-- ---------V AYIGGPVNNG EARIRADA-- --FRKALAAR AIPYDERLVA SG-QFTIDTG
sce5201   159 LGIDLSVGHM IDVHGRRN-- ---------I AYVGGPANNE EAKARADA-- --YWGVLAAR GLPLDERLFA VG-AFTIDSG
sce5204   125 LGLDLSVGHL VEDHGCQR-- ---------V AYIGGPPSNE EAEVRADA-- --YRRALADR SLRVDERLVA SG-SFTIDTG
sce6543   126 LDDKLVGVLQ MLNSKADH-- ---------F GPDERDVAEL LAAQAAVA-- --VQRTLLLE ERMIKLKLEH DLTIARDIQQ
sce8427   121 GELFSLSG-- -AAPGGAT-- ---------Q AAPGSATQDA AGASGSDE-- --AINEVRVQ LVALGDRLVQ LL-YDTAEKA
sce9114   28  IG-------- --PHAAIR-- ---------L RLPTRPAGHG EAELRVSG-- --YRSALAAR SIPCDERLVA HG-AFTIVTG
adeh0917  126 VRAAPGAAPR GIGLGEMRG- --------IVV DLPGLAHAGQ GAAVTADV-- --VPLARLLR GAPEGNALVL TAPVRDFAGD
sce0876   129 IGDYILEFQL TARAPRVP-- ---------I DPGGLHFEED KPTAIRKLGE LDPPRIAAKH LFTLMDLSRR LLSIEDATDR
sce1777   126 FRDKLVYNSK SNWWQNGPDE PTVVGVWGYL GKDGQILPDV QRAVDRTA-- --LLDLILPS FQKYGADKLQ VYYADSAEQP
sce2321   118 VGGEAAVAHV LDDHACKR-- ---------V AYISGPAHNE EAKLRAVA-- --YRRALEAR ALPYDERLFV YG-DFLIDGG

mxan4562  191 DVAPVRRAER RLVSAFYRGQ PISEIQEPID YGSSSGKGLV VISYSLGGLQ QQLASLEQDK RATVRATTLR MLGLGLGFVV
sce1779   184 AYSFLREVRR YATVNFVMFT DR-------- ---------F LRYVSGGGYG LLVRRGTPRS FNRVSN---- ----------
sce2617   190 RAAMRELLAR GVAFDAVVAA NDRMALAAIE VLKAEGRRIP EDVLVCGFDD VGIARFTKPS LTTVRQPIER LGGMAVSTVL
sce3610   78  TRGAAAALLR IDES------ ---------- ---------V GELEFSGVGN IAMKAITRTA MPSFCSA--- ----------
sce3741   79  TRGAAGMICR FRE------- ---------- ---------- GRLQGCGIGN VELMSHG-TR VQWLQSP--- ----------
sce5198   189 RAAMAEIIDR GVEIDAVVAA NDNMALGAIE LLKARGLHVP RDILVCGFDD VGVARYAKPS LTTVRQPIKR LGEIALETVV
sce5201   223 RVAIRDIIDR GVEVDAVVAA NDKMALGVIE LLAERGLRVP QDILVSGFDD AFIARFSKPS LTTVRQPMKR LGAQAMDTMM
sce5204   189 RAAMREIIGR GVAFDAVVAA NDNMALGAIE ELKAHGLSVP RDLPVCGFDD AVIARFAKPS LSTIRQPIKR IGARAVDIIV
sce6543   191 NILLRRLPRC PGYSLSAFSK PADDTGGDIY DVIRLDERVD MSPMLLLLAD AAGHGIGPAL SVTQFRAMLR IG--------
sce8427   182 SLKKQLEVAR AVQQMLVPSE D------VIE RPSLRLASHF QPAAECGGDW WTFHDLPDGR LLTVVGDVTG HG--------
sce9114   82  REAIREIIDR G--------- ---------- ---------- ---------- ---------- ---------- ----------
adeh0917  194 LCGYVVGAVT RDALARLLGE VSGRDGDYRP VVADAAGALT FSTPGIDAAA LAPVLAVPAG TPGTLERLEP AGGDPALAVR
sce0876   198 FDALCQLMVR EDFHGSMALA LRLRQNGALT ILSGPHRPFQ SLRREDSVAP PYISRRVLAA VQDTREPVLA GNLVSDSATV
sce1777   202 FVRVAPYLDM GGTFDKLYPG HNEKRWYDFF YPGLVESWSE WLKEPGGLES RPVQTTVTAP YDDAGGGGMI MTAFHPIWSK
sce2321   182 RRAMRELLGR RVEFDAVVAG NDNMALGAMD TLKAHGIDIP RDVLVCGFDD VAVSRVTRPS LTTVRHPIKE LGALAVETVV

mxan4562  271 LAG------- ---------- ---------- ---------- ---------- ---------- ---------- ----------
sce1779   232 ---------- ---------- ---------- ---------- ---------- ---------- ---------- ----------
sce2617   270 RMIDGEAVEA LQLARVELTQ RESCGCGYRA GDTFLQPVPS VRGFPRPIAD QRASLERALA SSVAIPSGAL DGWAGDLLAA
sce3610   119 ---------- ---------- ---------- ---------- ---------- ---------- ---------- ----------
sce3741   117 ---------- ---------- ---------- ---------- ---------- ---------- ---------- ----------
sce5198   269 RMLDGESVPE RNMQQIELTR RESCGCAYQA -IATRPLTNP PRLGAMSLGG RRDELERALE QAVGIPAASL DGWAGGLLSA
sce5201   303 RMLDGEVVED MALLDVELTC RESCGCRVQA SASLSPSAST PRDGPSLVAA RRESLETALR RSVAIPTGSL DGWPGELLSA
sce5204   269 RMIEGEPVPE YNLFPVELTL RESCGCGYQV EPAGAP-PSK AWSAPLPSAR QRDDLERLLE KSVAIPAGSL DGWAGLLLSA
sce6543   262 ---------- ---------- ---------- ---------- ---------- ---------- ---------- ----------
sce8427   247 ---------- ---------- ---------- ---------- ---------- ---------- ---------- ----------
sce9114   92  ---------- ---------- ---------- ---------- ---------- ---------- ---------- ----------
```

Figure S2: multiple sequence alignment of myxobacterial proteins assigned to COG2208

```
sce8427   247 ---------- ---------- ---------- ---------- ---------- ---------- ---------- ----------
sce9114   92  ---------- ---------- ---------- ---------- ---------- ---------- ---------- ----------
adeh0917  274 TQAATAAPRW ATLMLVPERV AYGKLTWLRV VLGLLYAG-- ---------- ---------- ---------- ----------
sce0876   278 ELTMS----- ---------- ---------- ---------- ---------- ---------- ---------- ----------
sce1777   282 GRDKFAGALG LDLTLAQLVA SIKDVRLANA GFAFLTQADG NVLAVNDLGV TTLRLRAEGG AGQSEGVGVL QRRLKDSSDP
sce2321   262 RQLRGERVPE CSLLPVELTR RESCGCGYYA ALPRAT-GAP PRREPLSLAA QREPLERALA RCVTVPAASR EAWPRALLSA

mxan4562  273 ---------- ---------- ---------- ---------- ---------- ---------- ---------- ----------
sce1779   232 ---------- ---------- ---------- ---------- ---------- ---------- ---------- ----------
sce2617   350 MEAELAGDEG RFLRALQALL DGARRQGVLL EEFQGVISLL RSRVQGSPVG G-----DAAL EQLWHAARIL IGSECVHVEG
sce3610   119 ---------- ---------- ---------- ---------- ---------- ---------- ---------- ----------
sce3741   117 ---------- ---------- ---------- ---------- ---------- ---------- ---------- ----------
sce5198   348 LEEELKGHDG CFLHVLERVL DTAGREGVFL DLFQGVVTLL RARLRRPVEE GGDAAVNEAL EQLWHACRVL IGSASVRVEG
sce5201   383 LEDELAGREN RFRHALEALL DAAGRAGAIL DHFQGVIDVL RERLGRPPSG GGGVALYDAL ERLWHVSRMV ISSASMRVEG
sce5204   348 LEEELAGTEG RFLRAVESLL DAAGREGAIL EHFQGMITLL REQFRRGHGA LRDTVLDEAL ERIWHASRIV IGSASVRAEG
sce6543   262 ---------- ---------- ---------- ---------- ---------- ---------- ---------- ----------
sce8427   247 ---------- ---------- ---------- ---------- ---------- ---------- ---------- ----------
sce9114   92  ---------- ---------- ---------- ---------- ---------- ---------- ---------- ----------
adeh0917  311 ---------- ---------- ---------- ---------- ---------- ---------- ---------- ----------
sce0876   282 ---------- ---------- ---------- ---------- ---------- ---------- ---------- ----------
sce1777   362 AVAMIALPSD DNVDYHERMI AG-------- EAYIIALQRL APVNSWAGPG ARIESQQWTL GFVVPKKELY ASLLASQRAI
sce2321   341 LEEELAGRED SFVTALQDLL DAAAREGVAL DQFQGAITLL RAKIGRTPGG ----APDDRL ERLWHTCRIL ISHASVRAEG

mxan4562  273 ---------- ---------- ---------- ---------- ---------- ---------- ---------- ----------
sce1779   232 ---------- ---------- ---------- ---------- ---------- ---------- ---------- ----------
sce2617   425 EQRLNVELAA IDVTYGARGF SACLSLPVLK GLLASELPRM RFSDVAVSLY D---DAQHAT MKPLFVMEDG HEVEVPAVSF
sce3610   119 ---------- ---------- ---------- ---------- ---------- ---------- ---------- ----------
sce3741   117 ---------- ---------- ---------- ---------- ---------- ---------- ---------- ----------
sce5198   428 RQRLDVEVAS IDLSWSGRWF STCLSLPLFQ RMLASEMPRL HFTRAAVSLY D---DAQRAT LKTLFLMEDG RAVAPPTESF
sce5201   463 ERRLTVELAA TYLSWSARSF STCLSLPSLK RVLASELPVL GISRAAVSLY DDAPEAQRGV MRSLFLMEDG REVDPPAASF
sce5204   428 QQRLNVELAS LYLSWSARSF STCLSLPILR RMMASELPRM QFSRVAVSLY DTAHDPQRAT MKALFLMEDG RELEPPAESF
sce6543   262 ---------- ---------- ---------- ---------- ---------- ---------- ---------- ----------
sce8427   247 ---------- ---------- ---------- ---------- ---------- ---------- ---------- ----------
sce9114   92  ---------- ---------- ---------- ---------- ---------- ---------- ---------- ----------
adeh0917  311 ---------- ---------- ---------- ---------- ---------- ---------- ---------- ----------
sce0876   282 ---------- ---------- ---------- ---------- ---------- ---------- ---------- ----------
sce1777   434 EESRTSIVTK QILVTIASFL ALMLGVYLVS RKMTGALVAL STGATRMREG D--------- ---------- ----------
sce2321   417 EKRLYLERAS VNLSWTGMFF STCLGMPTLR RTLAAELPKL DLTHVAISLF D---DPQRAT LQSLFLMENS REVEPPPASF

mxan4562  273 --------VL VAYQSRRITR PLGMLTGKVM QLAAGNLSAR AGTAQGAGRE VVTLGVVFNH MAERIKVLLE DVRAKAQLER
sce1779   232 ---------- ---------- ---------- ---------- ---------- ---------- ---------- ----------
sce2617   502 PAHLLAPPGF LSADERISRI VLPVAFGDEE RFGVAVLDHR ASGMIYEVLR LQLGSAVKAA ALHRQVVREV ELRERLEQER
sce3610   119 ---------- ---------- ---------- ---------- ---------- ---------- ---------- ----------
sce3741   117 ---------- ---------- ---------- ---------- ---------- ---------- ---------- ----------
sce5198   505 PSRSLAPPGF LSSSERTSVV VMPLTFGDTE YFGVMALNSG MNEMVYDALR LQIGSAVKTV ALHREMVRQV ELRERLEQES
sce5201   543 PARRLAPDGF LGGRERTTMI ALPVAFGDEE KFGVAVFSSG AHELIYDALR LQIGSATKAA ALHREMVRQV EARERLEQER
sce5204   508 PARRLAPEGF LGGRERTSMI ALPVAFGDEE KFGVAVLNSG ANEMVYDALR LQIGSAVKAA ALHWEVVRQV ELRERLEQEK
sce6543   262 ---------- ---------- ---------- ---------- ---------- ---------- ---------- ----------
sce8427   247 ---------- ---------- ---------- ---------- ---------- ---------- ---------- ----------
sce9114   92  ---------- ---------- ---------- ---------- ---------- ---------- ---------- ----------
adeh0917  311 -------VLL AAAGAGVAAV RQASRPLVDV SASMARVSRG DLTARVHAEY RDELGDLVRS FNVMVEEVAR SRDELQRTEA
sce0876   282 ---------- -----RDVMA LWVVACPLRV EEQMMNLLYV TLPPDFGSVE WLGLIALAAE VYQQAESAWE ARRHAQAHAA
sce1777   484 ---------- --YGVRIDVN SADEIGQLGV AFNDMASEIE AYTNNLEELV GERTRALEKA NEEISELNAK LAQENIRLGA
sce2321   494 AASCLAPPGF LSSSERWSVV AMPLTFGETE HYGIALVNGG ANEMVYDAAR LQIGSAMKAV SMHREIVRHV EVRERLEQER

mxan4562  346 EVSLARTVQE TLLPGREGVQ VGPLRIAGLV VTADACGGDW WFRAALDDRR -IVIGIGDVT GHGLSTSLVA TSATSGFASA
sce1779   232 ---------- ---------- ---------- ---------- ---------- ---------- ---------- ----------
sce2617   582 VRQESQVAAR IQTTLVPKQL SIEGLDLCAV MKPAAEVGGD YYDVIPANGG -GWLGIGDVA GHGLAAGLIM LMIQSMVSAL
sce3610   119 ---------- ---------- ---------- ---------- ---------- ---------- ---------- ----------
sce3741   117 ---------- ---------- ---------- ---------- ---------- ---------- ---------- ----------
sce5198   585 ERQETALAAR LQTTLVPERL HIEGLELSAA MTPAAEVGGD YYDVIATPEG -GWLGIGDVA GHGLAAGLVM LMIQSMVSAL
sce5201   623 VRQESVVAAR IQTTLVPERL SVAGLSLCAV MKPAAEVGGD YYDVLATADG -GWLGIGDVA GHGLAAGLVM LMIQSMVSAL
sce5204   588 VRQESVVAAR IQTTLVPAQL SVEGLELSAV MKPAAEVGGD YYDVLATDEG -GWLGIGDVA GHGLAAGLVM LMIQSMISAL
sce6543   263 LRLSADMDAL MHHVNQQLIE DLPNDRFITA FLGILDPTTH QLRFHAAGQG -PLLHYRAAE GQVVWRDPST VPLG------
sce8427   247 ------ISSA IITGAAKAAC DVVRTFAREA IGPAQLLRVM NCSIFEAGQQ -KFLMTCAAC IFDPAAGSLT LANAGHPFPY
sce9114   92  ---------- ---------- ---------- ---------- ---------- ---------- ---------- ----------
adeh0917  385 LRREVQIAHQ IQTAILPVSP AVPGYEVAAR MKPADDVGGD LYDILAFEDG -FWVLVGDVS GHGINSGLVM MMAQAAAYGA
sce0876   348 IERELEMAHQ IQRTFVPKRL EFKALDVALG FEPCRWVGGD YVDIVPMPDG RLMLAVADVC GKGLQAALIG SSVHTMVRAT
sce1777   553 ELNVARQLQL MVLPAPSELQ EIPGLDISGY MAPADEVGGD YYDVLRSDGL -VKIGIGDVT GHGLESGVLM LMVQTAVRTL
sce2321   574 VHQETLVAKH IQTTLVPAHL HVEGLELSAM MRPAAEVGGD YYDVLPTRDG -GWLGIGDVA GHGLVAGLVM LMIQSMVAAM

mxan4562  425 MTLREPSQVN AQMLITALNV TLANVGRGEH QMSSALAVID VSNGYIDYAA GAHPSPLVFN KRSGQIASLP ARG--PLLGA
sce1779   232 ---------- ---------- ---------- ---------- ---------- ---------- ---------- -------FIG
sce2617   661 TCNDPAASPA KVITALNKAI YKNVRDRLER DEHATLLLLR YERNGRVTFA GAHDDLVVCS ARTRRCGCIP SSGVWIGALP
sce3610   119 ---------- ---------- ---------- ---------- ---------- ---------- ---------- ------GIIG
```

Figure S2: multiple sequence alignment of myxobacterial proteins assigned to COG2208

```
sce2617     661 TCNDPAASPA KVITALNKAI YKNVRDRLER DEHATLLLLR YERNGRVTFA GAHDDLVVCS ARTRRCGCIP SSGVWIGALP
sce3610     119 ---------- ---------- ---------- ---------- ---------- ---------- ---------- ------GILG
sce3741     117 ---------- ---------- ---------- ---------- ---------- ---------- ---------- ------GILG
sce5198     664 TSRDPGASPS ELLAALNKAL YKNVRSRLKR DEHATLLLLR YERGGRVTFA GAHDDIIVCS ARTRRCVCIP SSGVWIGVLP
sce5201     702 TRRDPRASPA RVISAINAAL YENVRGRLMR DEHATLLLLR YERSGRVTFA GAHDDILVCR AKTRRCVCIP SSGVWIGTLP
sce5204     667 TRSDPRASPG DILSAVNGAV YENVRSRLKR DEHATLLLLR YERSGRVTFA GAHDDIIVCR AKTRRCVCIP SSGVWIGALP
sce6543     335 ---------- ---------- ---------- ---------- ---------- ---------- ---------- --------MF
sce8427     321 LVRNSAIRQL AAQGE----- ---------- ---------- ---------- ---------- ---------- ------PLGA
sce9114     92  ---------- ---------- ---------- ---------- ---------- ---------- ---------- ----------
adeh0917    464 IAEDPRCSPR HVIAAVNRVV HENVRRRMGR DDYLTLMAAR HLGDGRFVAA GAHQPIFVVR -GPGAVEVVD SPGPWVGLTA
sce0876     428 VDAGRGITGM MDRLN----- --AYLCEYLP EHSFVTMVAV ALDPATGRME CVNAGHPPAL VATAEGGLRH LQAAVNPALG
sce1777     632 LAANERDPKK FLSIVN--KV IYQNIQRIRS DKNLSLTLLD YSDGELQLTG QHEDLIIVRK DGTLDRVETM GLGLPIGIDL
sce2321     653 TRRDPGARPG EVVSALNRAI YDNIRDRLKR DEHATLLLLR YERGGRVTFA GAHDDIIVCR ARTRRSECIP SSGVWVGVLP

mxan4562    503 SVESQFTSRQ AQLRPGDVVV WYTDGLTEAR DNAGKLYGTQ RLAAAVQAHA HL----SAEA LRDAVLADAR AFSAGQPQRD
sce1779     236 LRIKPFHEFE LPFEEDDVLA LYTDGMFEAQ DASGEIYSVQ RLNEIVVKHS EE----PVQV ILDRCIEDYT RFR--VADAD
sce2617     741 EIGALTRDAE LYLGDGDVLV LYSDGVTEAR NAHHEQFGLE RLCAVIEAVQ TE----PVGV IRDRILDAVE GWC--PSPDD
sce3610     124 RRVRRLRSFR FPISPGDVVL LHSDGISSG- ------FDLG -------AFR DL----DPEE MARRIVDAHK ------KSFD
sce3741     122 ASLSKVRIFD AQLARGDRLV VFTDGVSPR- ------FDLS -------LFR GM----KAAD ACRALMSRHR ------RSHD
sce5198     744 VIDEVTRDAQ FVLEDGDVLV LYSDGVTEAR NAHREQFGLE RLCTAIEAVQ TA----PVEA IRDRILREVE GWC--PSPDD
sce5201     782 SINELTRDAE FVLEDGDVLV LYSDGVTEAR DAHHEQFGLE RLCTTIEAVQ TE----SVVV IRDRILKEVE GWC--PSPDD
sce5204     747 AIDAMTRDAE FVLEDGDVLV LYSDGVTEAR NAHHEQFGLE RLCSTIESSQ SA----PAEA IRDRILGEVE AWC--PSPDD
sce6543     338 KDLDLPPTPP LVMAPGDLLV LLTDGFYEYQ DAAGQVFGKE RVGDLIHRLH AR----PTSD ILGALLAEVR RFAGSAKQID
sce8427     340 ASSAEYTSST IALEGGDALL WFTDGVTECE NESSEQFSEK RLRSLFQSVA AG----PPEE ARDAIVAAVS RFRGERALDD
sce9114     92  ---------- IVLEDGDVLV LYSDGVTEAR NAHQEWYGLE RLCAAIEAVQ AA----PVDA IRDRILEQVE GWC--PSLDD
adeh0917    543 GVPPGVTEYE FQVGPGELLC LVTDGMLEAP AAGGELFGED RLAQVLGDLH GA----SAPQ ALGTVFSAVE AFA--ASQED
sce0876     501 VGPVPMEAHQ SVLAPGEVLA MYTDGLTELR NSSKQMLGLH ALGTGFAKLC ATSAGRPVAS IADKLTHMLE EFRGDQLPED
sce1777     710 DISDFIGSIE VKLQPGDVVT LFSDGITEAE DSSEEQYGID RLCQVISRNH QR----TSRE IKDAIIEDVL AHVGYNKIHD
sce2321     733 SIDATTRDAE LLLEDGDLLV LYSDGVTEAR NAHREQFGLS RLCATVEAAQ DA----PVDD IRDRILREVD GWC--PSPDD

mxan4562    579 DITVVVAEFS PAA--
sce1779     310 DITLIILRRC A----
sce2617     815 DITIVVARYR APE--
sce3610     180 DATCVVAVVR DRGAA
sce3741     178 DSTVLIADIE P----
sce5198     818 DITVVVARYR APA--
sce5201     856 DITIVVARYR APRQP
sce5204     821 DITVVVARYS APRTL
sce6543     414 DLTALLVKRD A----
sce8427     416 DVTLVVARVS -----
sce9114     157 DITILVARYR APR--
adeh0917    617 DMTTVVLRRK NDDR-
sce0876     581 DRAFLLARRT -----
sce1777     786 DITVLVIKRV -----
sce2321     807 DITIVVGRYR APR--
```
